# Enzymatic and Chemoenzymatic Three‐Step Cascades for the Synthesis of Stereochemically Complementary Trisubstituted Tetrahydroisoquinolines

**DOI:** 10.1002/anie.201705855

**Published:** 2017-09-06

**Authors:** Vanessa Erdmann, Benjamin R. Lichman, Jianxiong Zhao, Robert C. Simon, Wolfgang Kroutil, John M. Ward, Helen C. Hailes, Dörte Rother

**Affiliations:** ^1^ IBG-1: Biotechnology Forschungszentrum Jülich GmbH 52425 Jülich Germany; ^2^ John Innes Centre Norwich Research Park Norwich NR4 7UH UK; ^3^ Department of Chemistry University College London London WC1H 0AJ UK; ^4^ Department of Biochemical Engineering University College London London WC1E 6BT UK; ^5^ Roche-Diagnostics GmbH DOZCBE 82377 Penzberg Germany; ^6^ Department of Chemistry University of Graz 8010 Graz Austria

**Keywords:** asymmetric catalysis, biocatalysis, chemoenzymatic cascades, norcoclaurine synthase, transaminases

## Abstract

Chemoenzymatic and enzymatic cascade reactions enable the synthesis of complex stereocomplementary 1,3,4‐trisubstituted tetrahydroisoquinolines (THIQs) with three chiral centers in a step‐efficient and selective manner without intermediate purification. The cascade employs inexpensive substrates (3‐hydroxybenzaldehyde and pyruvate), and involves a carboligation step, a subsequent transamination, and finally a Pictet–Spengler reaction with a carbonyl cosubstrate. Appropriate selection of the carboligase and transaminase enzymes enabled the biocatalytic formation of (1*R*,2*S*)‐metaraminol. Subsequent cyclization catalyzed either enzymatically by a norcoclaurine synthase or chemically by phosphate resulted in opposite stereoselectivities in the products at the C1 position, thus providing access to both orientations of the THIQ C1 substituent. This highlights the importance of selecting from both chemo‐ and biocatalysts for optimal results.

The tetrahydroisoquinoline (THIQ) moiety is a “privileged scaffold”,^1^ and as such, it is found in numerous bioactive natural products (e.g., noscapine,^2^ dioncophyllines^3^) and synthetic pharmaceutical drugs (e.g., solifenacin^4^). THIQ‐containing compounds demonstrate a wide range of bioactivities, including antitumor,^2^ antiparasitic,^3^ and anticholinergic^4^ properties.

Naturally derived THIQs may be obtained by extraction or fermentation,^5^ but in many cases, only small quantities are available, typically as one component of a mixture. Chemical syntheses are a frequent source of these compounds, but complex multistep asymmetric routes that give high stereoselectivities can be challenging, including at scale, and are often dependent on the use of toxic or environmentally harmful chemicals.^6^ Therefore, novel synthetic routes towards THIQs are of significant interest.

In vitro biocatalysis provides a viable method of producing complex THIQs in high stereoselectivities and under mild conditions.^7^, ^8^, ^9^ Previous studies have shown norcoclaurine synthase (NCS), the Pictet–Spenglerase from plant benzylisoquinoline alkaloid biosynthesis,^10^, ^11^ to be a versatile biocatalyst. NCS can convert 3‐hydroxyphenethylamines (e.g., dopamine) and a carbonyl cosubstrate into a (1*S*)‐THIQ with high stereoselectivities.^12^‐^15^ The enzyme has demonstrated a remarkably wide carbonyl cosubstrate tolerance, accepting a variety of aldehydes and ketones.^13^‐^20^

NCS has also been successfully employed in chemoenzymatic cascades.^12^, ^15^, ^16^, ^21^ As an alternative to NCS, inorganic phosphate can be used to catalyze the Pictet–Spengler reaction (PSR), typically forming racemic THIQs under aqueous conditions.^22^ This can be successfully combined with enzyme steps.^21^

Modular biocatalytic cascades^23^ for the stereoselective production of pharmaceutical compounds are exemplified by the work conducted on phenylpropanolamines.^24^, ^25^, ^26^ All four possible isomers of nor(pseudo)ephedrine are available from simple starting materials by one‐pot two‐enzyme cascades when appropriate biocatalysts are selected.^26^ Phenylpropanolamines with an additional 3‐hydroxy group on the aromatic ring have been considered to be suitable substrates for cascades including PSRs catalyzed by NCS or phosphate. Indeed, a previous screen of *Coptis japonica* NCS (*Cj*NCS2) had suggested that (1*R*,2*S*)‐2‐amino‐1‐(3‐hydroxyphenyl)propan‐1‐ol (also referred to as metaraminol) was accepted as a substrate, and gave the corresponding product in low yield.^13^ With this in mind, two three‐step cascades, using either three enzymes or two enzymes and one non‐enzymatic step, were designed for the stereoselective formation of 1,3,4‐trisubstituted THIQs (Scheme 1).

**Scheme 1:**
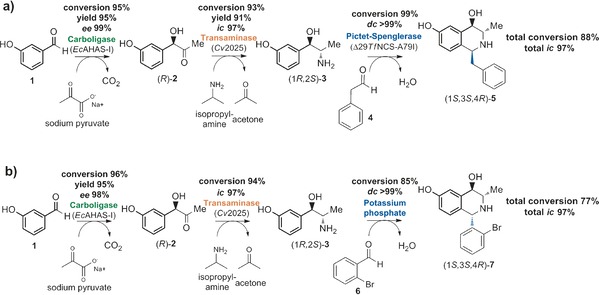
a) Enzymatic and b) chemoenzymatic three‐step cascades to isoquinoline derivatives. Reaction conditions: step 1: 100 mm HEPES, pH 7.5, 5 mm MgCl_2_, 0.1 mm ThDP, 0.05 mm FAD, 10 mm **1**, 20 mm sodium pyruvate, 2.5 % DMSO (v/v), 0.5 mg mL^−1^ acetohydroxy acid synthase I from *Escherichia coli* (*Ec*AHAS‐I), 30 °C, 750 rpm; step 2: 0.2 mm PLP, 100 mm IPA, 3.1 % DMSO (v/v), 3 mg mL^−1^ transaminase from *Chromobacterium violaceum (Cv*2025), 30 °C, 750 rpm; step 3 a: 9.5 mm **4**, 2.5 % DMSO (v/v), 0.5 mg mL^−1^ norcoclaurine synthase variant from *Thalictrum flavum* (Δ*Tf*NCS‐A79I), 37 °C, 750 rpm; step 3 b: 10 mm **6**, 200 mm potassium phosphate buffer, pH 7, 50 °C, 750 rpm. For more detailed information on the set‐up, see Section S2.

Herein, we present the first three‐step (chemo)enzymatic cascades for the stereoselective formation of 1,3,4‐trisubstituted THIQs from inexpensive starting materials. The cascades begin with a carboligation using 3‐hydroxybenzaldehyde (**1**; Scheme 1) and pyruvate to form acyloin **2**; subsequent transamination gives rise to 2‐amino‐1‐(3‐hydroxyphenyl)propan‐1‐ol (**3**), which can then undergo a PSR with a carbonyl cosubstrate. Most importantly, NCS and phosphate catalysis of the PSR step result in opposite stereoselectivities, thus providing access to both orientations of the THIQ C1 substituent, which highlights the importance of selecting from both chemo‐ and biocatalysts.

The first requirement for chemoenzymatic cascades towards complex trisubstituted THIQs was the development of a two‐step cascade for the production of the amino alcohol (1*R*,2*S*)‐**3** (Scheme 1). The initial step in the cascade was the carboligation of 3‐hydroxybenzaldehyde (**1**) and acetaldehyde (after decarboxylation from pyruvate) to form (*R*)‐1‐hydroxy‐1‐(3‐hydroxyphenyl)propan‐2‐one ((*R*)‐**2**). This was catalyzed by the ThDP‐dependent acetohydroxy acid synthase I from *Escherichia coli* (*Ec*AHAS‐I),^27^ which was also capable of catalyzing the decarboxylation of pyruvate to acetaldehyde prior to carboligation in the same active site. After 1 hour incubation, the reaction was complete with excellent conversions (conversion 95 %, yield 95±2 %) and excellent enantioselectivities (*ee* 99 %). To form (1*R*,2*S*)‐**3**, the hydroxyketone intermediate (*R*)‐**2** was immediately subjected to transamination with the transaminase from *Chromobacterium violaceum* (*Cv*2025)^28^ using isopropylamine as the amine donor.

This step was complete in 8 hours and provided excellent conversion (conversion 93±1 %, yield 91±3 %) and excellent isomeric content of one isomer over all possible isomers (97±1 %; for the *ic* definition, see the Supporting Information, Section S5). The enzymes described for this two‐step cascade have been valuable catalysts for cascade reactions with unsubstituted aromatic aldehydes and hydroxyketones.^25^, ^29^ Here, they also proved to be suitable catalysts for a substituted benzaldehyde, yielding biocatalytic access to (1*R*,2*S*)‐**3** (metaraminol), an α_1_ adrenergic receptor agonist that acts as a vasoconstrictor.^30^

Compound **3** was then used for a subsequent PSR. A vital feature of **3** is the presence of the *meta*‐hydroxy group, which is mechanistically required for both the mild phosphate‐catalyzed and the enzyme‐catalyzed PSR.^22^, ^31^ The carbonyl cosubstrate phenylacetaldehyde (**4**) was selected as a representative aldehyde for the proof‐of‐principle reactions using NCSs. NCS wild‐type enzymes from *Coptis japonica* and *Thalictrum flavum* and available variants were screened against **3** and **4**, and the formation of products was monitored by HPLC analysis (see the Supporting Information, Section S6 for details). The variant *Tf*NCS‐A79I was found to produce the largest quantity of product. In a recent study, this variant had also demonstrated increased conversion compared to the wild‐type enzyme for reactions with dopamine and ketones.^18^ Therefore, *Tf*NCS‐A79I was selected for a preparative (50 μmol) biotransformation reaction for product identification, using **4** and (1*R*,2*S*)‐**3** from a commercial source. The THIQ product (1*S*,3*S*,4*R*)‐1‐benzyl‐3‐methyl‐1,2,3,4‐tetrahydroisoquinoline‐4,6‐diol (**5**) was isolated in excellent yield (92 %, **5**⋅HCl) and purity (*de* 99 %). Analysis by both HPLC on a chiral stationary phase and two‐dimensional NMR spectroscopy confirmed the presence of (1*S*,3*S*,4*R*)‐**5** (see Section S4.1).

With the isolated product **5** and analytical data for the isolated stereoisomer as well as a suitable separation method for all possible stereoisomers in hand (Section S3), the entire three‐step three‐enzyme cascade was demonstrated. As described above and shown in Scheme 1, (1*R*,2*S*)‐**3** was formed biocatalytically from 3‐hydroxybenzaldehyde (**1**), pyruvate (**2**), and isopropylamine in two enzyme‐catalyzed steps. Subsequently, *Tf*NCS‐A79I and **4** were added. The conversions and stereoselectivities for this last step were excellent (conversion 99±1 %, *dc* >99 %). Overall the cascade conversion of **1** into (1*S*,3*S*,4*R*)‐**5** was achieved in 88 % yield (calculated from substrate depletion; Section S8 and Figure 39), with excellent optical purities after each step (Scheme 1 a; only one product, the desired one, was identified by HPLC and GC‐TOF‐MS analysis; see Section S3 and Figures S8 d and S12). The final *ic* value was >97 %. This formation of a complex trisubstituted THIQ, with the highly selective installation of three chiral centers, high reaction yields, and no intermediate purification, is a clear demonstration of the power of biocatalytic cascades for the production of complex molecules. What is crucial to note in this case is that NCS is a particularly promiscuous enzyme with respect to the carbonyl cosubstrate, with no observed loss in stereoselectivity,^13^, ^14^, ^15^ and therefore, this cascade can be easily adapted to produce a wide variety of 1*S*‐configured THIQs.

The PSR of *meta*‐hydroxyphenylethylamines and aldehydes can also be very effectively catalyzed by inorganic phosphate.^22^ In the initial screening with (1*R*,2*S*)‐**3** and **4** (which was selected for the three‐step enzyme cascade), phosphate catalysis was observed to give rise to three products (Section S3.4), where one of the minor products was (1*S*,3*S*,4*R*)‐**5**. To probe the stereoselectivity of the phosphate‐catalyzed PSR, various other aldehydes were examined. A number of carbonyl cosubstrates were screened, and the product selectivities were monitored (Section S7). The use of more sterically demanding aldehydes increased the reaction selectivities immensely. Notably, the aldehyde 2‐bromobenzaldehyde (**6**) provided the best stereoselectivity in the product. (Although priority naming rules dictate the major product here to be an (1*S*)‐THIQ, the configuration of the C1 stereocenter is opposite to that of THIQs produced by NCS.) To isolate and characterize the product (1*S*,3*S*,4*R*)‐1‐(2‐bromophenyl)‐1,2,3,4‐tetrahydroisoquinoline‐4,6‐diol (**7**), this reaction was scaled up (20 μmol), and the product was purified by extraction and recrystallization to give **7**⋅HCl in 54 % yield (97 % *de*) after isolation. Analysis by both HPLC on a chiral stationary phase and two‐dimensional NMR spectroscopy confirmed the compound to be (1*S*,3*S*,4*R*)‐**7** (97 % *de* before recrystallization, 99 % *de* after recrystallization; see Section 4.2).

The three‐step chemoenzymatic cascade was then performed (Scheme 1 b). After the amino alcohol (1*R*,2*S*)‐**3** had been formed in two enzyme‐catalyzed steps as described above, the enzymes were removed by ultrafiltration, and potassium phosphate and **6** were added. This final step proceeded with high conversions (85 %, 24 h) and excellent stereoselectivities (*dc* 99 %; Section 3.5 and Figure S9 c). The major product of the cascade was the anticipated (1*S*,3*S*,4*R*)‐1‐(2‐bromophenyl)‐3‐methyl‐1,2,3,4‐tetrahydroisoquinoline‐4,6‐diol (**7**). Minor side products were also formed; these are believed to be the *ortho*‐cyclized products 1‐(2‐bromophenyl)‐3‐methyl‐1,2,3,4‐tetra‐hydroisoquinoline‐4,8‐diol (<4 %, see Sections S3.5 and 4.3; validated by GC‐TOF‐MS, HPLC, and NMR analysis by correlation to the non‐cascade KPi reaction products).^16^ Overall, the conversion of the cascade from **1** to (1*S*,3*S*,4*R*)‐**7** was 77 %, based on the substrate depletion of each step. Again, the final *ic* value was excellent (>97 %). The enzymatic synthesis of the opposite stereoisomer (1*R*,3*S*,4*R*)‐**7** was not possible as the NCS enzymes screened did not accept the sterically demanding aldehyde 2‐bromobenzaldehyde as a substrate.

The high stereoselectivity of the phosphate‐catalyzed PSR reaction with (1*R*,2*S*)‐**3** can be explained by the three‐dimensional structure of the phenethylamine substrate. In the transition state of the phosphate PSR cyclization step, the molecule likely adopts a pseudo‐chair conformation with the aldehyde R group occupying a pseudo‐equatorial orientation (Figure 1). In the phosphate‐catalyzed reaction, the favored transition state also has the methyl and hydroxy substituents in pseudo‐equatorial positions. Bulkier aldehyde substituents increase the stereoselectivity, presumably owing to increased steric hindrance and an increase in the energy of the less favored transition state (in which the methyl and hydroxy groups are pseudo‐axial). This is an example of substrate‐controlled diastereoselectivity, in which the existing stereocenters determine the installed stereocenter. The enzymes *Ec*AHAS‐I and *Cv*2025 thus establish the stereocenters in the amino alcohol (**3**), which in turn determine the THIQ C1 stereocenter. It is pertinent to note that the THIQ C1 configuration available with the phosphate‐catalyzed method here is opposite to that obtained with NCS. Thus the phosphate‐mediated PSR provides direct access to “unnatural” THIQ stereoisomers, without the requirement for discovering or engineering a novel NCS with inverted stereoselectivity.

**Figure 1:**
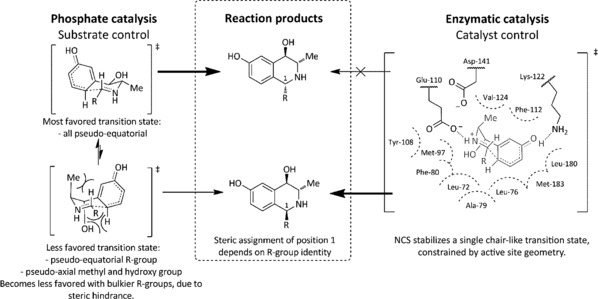
Rationale for the reaction selectivity.

The substrate‐controlled outcome of the phosphate‐catalyzed reaction contrasts with the catalyst‐controlled stereoselectivity in the enzyme‐catalyzed reactions. In contrast to some transaminases and imine reductases,^32^, ^33^ for which changing stereopreference, depending on the selected substrates and cosubstrates, has been described, NCS can only stabilize one transition state owing to the arrangement of the active‐site side chains, giving rise to only (1*S*)‐THIQs (Figure 1).^13^, ^14^, ^34^ In this transition state, the aldehyde‐derived substituent is pseudo‐equatorial and oriented towards the active‐site entrance while the methyl and hydroxy substituents are in pseudo‐axial positions. In this scenario, the constraints of the biocatalyst active‐site architecture overwhelm any conformational preferences of the intermediate.

Whereas the phosphate‐ and enzyme‐catalyzed reactions provide access to the opposite configurations at C1 in the PSR with (1*R*,2*S*)‐**3**, the aldehyde cosubstrates required for maximum conversion and stereoselectivity differ. Aldehyde **4**, an excellent substrate for NCS, seems to be “insufficiently bulky” to provide good selectivity in the phosphate‐catalyzed variant. Conversely, more sterically demanding 2‐bromobenzaldehyde (**6**) is an excellent cosubstrate in the stereoselective phosphate‐catalyzed reaction; however, it is not accepted by NCS wild‐type enzymes or variants thereof. Future catalyst engineering of either phosphate or NCS would help increase the overlap of suitable aldehyde cosubstrates. Synthesizing bulkier phosphate catalysts to improve the stereoselectivity for other aldehydes^35^ and enzyme engineering of NCS to improve substrate tolerance towards bulkier aldehydes^14^ could expand the range of this system further.

A crucial feature of the cascade systems for the production of phenylpropanolamines is enzyme modularity, which principally enables the construction of all four isomers by carboligases and transaminases with complementary stereoselectivity (see Section S6). Such an approach will be taken in future work with all stereoisomers of the 3‐hydroxy‐substituted hydroxyketone intermediate **3**, and these isomers will be examined with phosphate and NCS in PSRs to form novel 1,3,4‐trisubstituted THIQs. Furthermore, in a successful substrate screening with aldehydes, further reaction partners were identified to create a large THIQ library (see Section S7).

In conclusion, we have demonstrated the applicability of chemoenzymatic and enzymatic cascades for the formation of complex chiral compounds bearing three chiral centers from low‐cost starting materials in a rapid and selective manner without intermediate purification. The stereocomplementary PSRs resulting from chemical or enzymatic catalysis highlight how (aqueous‐based) chemical processes can work in partnership with biocatalysis. The diversity of THIQs accessible by biocatalysis has now been expanded significantly.

## Conflict of interest

The authors declare no conflict of interest.

## Supporting information

As a service to our authors and readers, this journal provides supporting information supplied by the authors. Such materials are peer reviewed and may be re‐organized for online delivery, but are not copy‐edited or typeset. Technical support issues arising from supporting information (other than missing files) should be addressed to the authors.

Supplementary
