# Supplementary material for: Enzymatic and Chemoenzymatic Three‐Step Cascades for the Synthesis of Stereochemically Complementary Trisubstituted Tetrahydroisoquinolines
Source: Angew Chem Int Ed Engl. 2017 Sep 6;56(41):12503–7. doi: 10.1002/anie.201705855 (PMC5658969; doi:10.1002/anie.201705855)
Supplement: Supplementary file 1 — Supplementary [file ANIE-56-12503-s001.pdf]

Angewandte Chemie
Eine Zeitschrift der Gesellschaft Deutscher Chemiker

Supporting Information

Enzymatic and Chemoenzymatic Three-Step Cascades for the Synthesis of Stereochemically Complementary Trisubstituted Tetrahydroisoquinolines

*Vanessa Erdmann, Benjamin R. Lichman, Jianxiong Zhao, Robert C. Simon, Wolfgang Kroutil, John M. Ward, Helen C. Hailes, and Dörte Rother**

anie_201705855_sm_miscellaneous_information.pdf

Supplementary Information

Table of content

1. Transformation, cultivation and purification 3
   1.1 Expression and purification of the norcoclaurine synthase from *Thalictrum flavum* variant (△29*Tf*NCS-A79I) for reference compound synthesis (UCL London) 3
   1.2 Expression and purification of enzymes for biotransformation (Forschungszentrum Jülich GmbH) 4
   1.3 Nucleotide and amino acid sequences of all biocatalysts 8
2. Reaction conditions 10
   2.1 Enzymatic 3-step cascade reaction 10
   2.2 Chemoenzymatic 3-step cascade reaction 12
3. Instrumental analysis 12
   3.1 Sample preparation of carboligation measurements (step 1) - HPLC 12
   3.2 Sample preparation of transamination measurements (step 2) - HPLC 13
   3.3 Sample preparation of transamination measurements - SFC 14
   3.4 Sample preparation of Pictet-Spengler condensation (step 3) catalysed by NCS - HPLC 15
   3.5 Sample preparation of Pictet-Spengler reaction catalysed by potassium phosphate - HPLC 16
   3.6 Product identification by GC-TOF-MS 17
4. Synthesis of reference compounds 25
   4.1 (1*S*,3*S*,4*R*)-1-benzyl-3-methyl-1,2,3,4-tetrahydroisoquinoline-4,6-diol synthesis 25
   4.2 (1*S*,3*S*,4*R*)-1-(2-bromophenyl)-3-methyl-1,2,3,4-tetrahydroisoquinoline-4,6-diol synthesis 30
   4.3 (1*S*,3*S*,4*R*)-1-(2-bromophenyl)-3-methyl-1,2,3,4-tetrahydroisoquinoline-4,6-diol synthesis under the 3-step reaction cascade conditions (3.5) to identify minor isomers formed 35
5. Calculation of conversion, yield, *ee*, *de*, *dc* and *ic* values 40

6. Conversion of 2-amino-1-(3-hydroxyphenyl)propan-1-ol isomers by norcoclaurine synthase variants and phosphate (step 3) 41
7. Substrate screening for phosphate catalysed Pictet-Spengler reaction (step 3) 43
8. Time curve of the enzymatic 3-step cascade reaction to (1*S*,3*S*,4*R*)-1-benzyl-3-methyl-1,2,3,4-tetrahydroisoquinoline-4,6-diol (step 1-3) 44
9. Literature 46

1. Transformation, cultivation and purification

1.1 Expression and purification of the norcoclaurine synthase from *Thalictrum flavum* variant (△29*Tf*NCS-A79I) for reference compound synthesis (UCL London)

Expression of △29*Tf*NCS-A79I[1]: A pJ411 plasmid containing a codon optimised Δ29*Tf*NCS-A79I was purchased from DNA2.0 and *E. coli* BL21 (DE3) cells were transformed with these plasmids by a standard heat-shock protocol. The transformed cells were stored as glycerol stocks at -80 °C. An aliquot from the frozen stock was inoculated into 20 mL of TB medium (containing 50 µg/mL kanamycin). Starter cultures were incubated overnight at 37 °C, shaking at 250 rpm. A 4 mL sample of the overnight starter culture was added to 100 mL of TB medium. The expression cultures were incubated for 2 hours at 37 °C, followed by 1 hour at 25 °C, whilst shaking at 250 rpm. Expression was induced by the addition of 100 µL of 500 mM IPTG. Cultures were incubated for a further 3 hours at 25 °C prior to harvesting, whilst shaking at 250 rpm. Cells were harvested by centrifugation at 10,000 g for 10 mins at 4 °C. Supernatant was removed and the cells stored at -20°C until purification.

Purification of △29*Tf*NCS-A79I (10 mL scale): Cell pellets were thawed and suspended in BugBuster (10 mL), a detergent based lysis agent for *E. coli* (Novagen). The insoluble portion of the lysate was pelleted by centrifugation at 10,000 x g for 30 minutes at 4 °C. The supernatant was removed and filtered through a glass fibre prefilter and 0.2 µm cellulose acetate syringe filter. An empty and clean PD-10 column (GE) was charged with Ni-NTA (2 mL). The column was washed with distilled water (10 mL), followed by binding buffer (0.1 M HEPES, 20 mM imidazole, 100 mM NaCl, pH 7.5; 10 mL). The filtered supernatant was passed through the Ni-NTA column, and the column was then washed with binding buffer (10 mL) followed by wash buffer (0.1 M HEPES, 40 mM imidazole, 100 mM NaCl, pH 7.5; 20 mL). The bound protein was then eluted with elution buffer (0.1 M HEPES, 500 mM imidazole, 100 mM NaCl, pH 7.5; 5 mL). The eluent containing pure enzyme was buffer exchanged into 0.1 M HEPES pH 7.5, using a PD-10 column. Glycerol was added (10% v/v) and the concentration of the protein determined by absorbance at 280 nm. The protein was divided into 0.5 mL samples and frozen in liquid nitrogen. The purified protein was stored at -80 °C.[2]

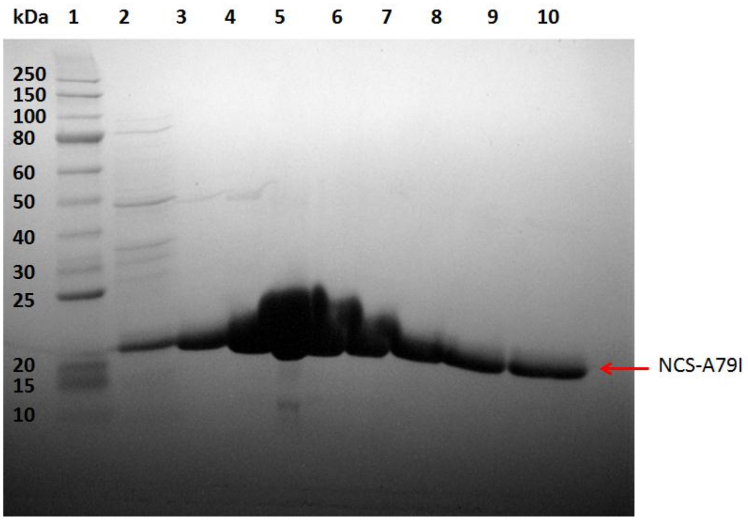

Figure 1 SDS-PAGE of △29*Tf*NCS-A79I. 1: protein ladder; 2: flow through with wash buffer (containing 20 mM imidazole); 3: flow through with wash buffer (containing 40 mM imidazole); 4-10: purified protein in different fractions.

According to SDS-gel estimation the protein purity was >98% as no further bands were obvious. The concentration of prepared △29*Tf*NCS-A79I was measured using Nanodrop software at 280 nm in triplicate. Parameters calculated from ProtParam (ref see below): MW= 21195.39. ε = 19940 $M^{-1}cm^{-1}$. The average concentration of △29*Tf*NCS-A79I was 2.4 mg/mL.

1.2 Expression and purification of enzymes for biotransformation (Forschungszentrum Jülich GmbH)

1.2.1 Acetohydroxy acid synthase I from *Escherichia coli* (*Ec*AHAS-I)

Cells containing a plasmid encoding for *Ec*AHAS-I as described in SI-1.3.1 were produced in a 1 L-DASGIP cultivation system in 0.645 L autoinduction medium and 0.450 L feed solution at 20 °C, 1200 rpm, pH 7, 6 – 18 sL/h oxygen supply (30% dissolved oxygen concentration) and 1 mL/L anti foam 204. The 33% feed solution was added steadily (6.75 mL/h, table 1).

Table 1 Autoinduction medium by Studier and used feed solution.

**Media component**	**Autoinduction medium**	**Feed solution (33%)**
Peptone	12 g/L	51.1 g/L
Yeast extract	24 g/L	102.2 g/L
**Further components**		
Potassium phosphate buffer 1M, pH 7.0	90 mL/L	-
Lactose	2 g/L	16.7 g/L
Glucose	0.5 g/L	-
Glycerol	5 mL/L	41.9 g/L

The *E. coli* BL21 (DE3) cells with the overexpressed *Ec*AHAS-I were diluted in equilibration buffer pH 7.6 and resuspended on ice. The cells were opened by sonification (UP 200S Dr. Hielscher, S14 D-sonotrode, cycle 0.5, amplitude 70%) and cell debris removed by centrifugation (JA-20, 19000 rpm, 4 °C, 35 min). *Ec*AHAS-I was purified from crude cell extract (column material: Ni-NTA superflow, Qiagen) with an automated Äkta purifier system (GE Healthcare). Afterwards the proteins were desalted and rebuffered by size exclusion chromatography (Sephadex G25 column) and the purification success tested by SDS-PAGE. *Ec*AHAS-I consists of a catalytic subunit (62.5 kDa) and a regulatory subunit (11.1 kDa), which both need to be present in order to maintain maximal enzyme activity.[3–5] Enzymes were lyophilised (Christ, LT105) and stored at -20 °C (330 mg lyophilisate, 0.4 mg protein/mg lyophilisate (Bradford)).

Table 2 Purification buffers (pH 7.6) used for the Ni-NTA purification and desalting of *Ec*AHAS-I by G25-column.

	HEPES [mM], pH 7.6	KCl [mM]	FAD [mM]	Imidazole [mM]
Equilibration buffer	100	500	0.01	20
Wash buffer	100	500	0.01	50
Elution buffer	100	500	0.01	300
Desalting buffer	10	0	0.02	0

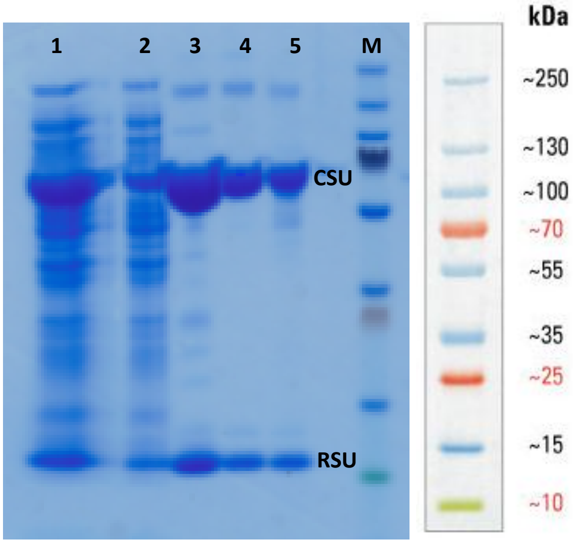

Figure 2 SDS-PAGE of *Ec*AHAS-I purification by automated Äkta purifier system, GE Healthcare. 1: crude cell extract, 2: flow through, 3: wash fraction, 4: elution fraction, 5: desalting fraction (AHAS-I after purification), M: protein ladder (PageRuler Plus Prestained, Thermo Fisher Scientific), CSU: catalytic subunit, RSU: regulatory subunit.

According to SDS-gel estimation the protein purity was >95%.

1.2.2 Transaminase from *Chromobacterium violaceum* (*Cv*2025)

Cells containing a plasmid encoding for transaminase *Cv*2025 were cultivated as described elsewhere.[6–8] *Cv*2025 was purified by Ni-NTA purification from crude cell extract (column material: Ni-NTA superflow, Qiagen, automated Äkta purifier system, GE Healthcare) and desalted and rebuffered by size exclusion chromatography (Sephadex G25 column). The enzymes were lyophilised (Christ, LT105, ~1 mg protein/mg lyophilisate, Bradford, purity 90%, protein content 70% according to SDS-PAGE) and stored at -20 °C.[9]

Table 3 Purification buffers (pH 7.5) used for the Ni-NTA purification and desalting of *Cv*2025 by Sephadex G25-column.

	HEPES [mM], pH 7.5	PLP [mM]	Imidazole [mM]
Equilibration buffer	100	0.2	0
Wash buffer	100	0.2	25
Elution buffer	100	0.2	250
Desalting buffer	10	0.2	0

1.2.3 Norcoclaurine synthase variant from *Thalictrum flavum* (Δ29*Tf*NCS-A79I)

Expression of Δ29*Tf*NCS-A79I: *E. coli* BL21 (DE3) was transformed with a pJ411 plasmid containing codon optimised Δ29*Tf*NCS-A79I (SI-1.3.3) by a standard heat-shock protocol. The

grown colonies were used for cultivation of the cells in TB medium supplied with kanamycin and induced by 500 µM IPTG after 3-4 h (90 rpm, 20 °C). Cells were harvested (16.8 g/L) and used for purification of the overexpressed Δ29*Tf*NCS-A79I. Overexpression was controlled by SDS-PAGE (20.98 kDa).

Purification of Δ29*Tf*NCS-A79I (50 mL scale): cells were solved in HEPES-buffer, pH 7.5 and opened by sonification (UP 200S Dr. Hielscher, S3-sonotrode 12x12 sec. with a break of 40 sec., cycle 0.5, amplitude: 40%). The cell debris was removed by centrifugation (20.000 rpm, 4 °C, 45 min) and the supernatant was filtered (syringe filters, polypropylene 0.45 µm, GE). The Δ29*Tf*NCS-A79I was purified from crude cell extract (column material: Ni-NTA superflow, Qiagen) with an automated Äkta purifier system (GE Healthcare). Subsequently, the proteins were desalted and rebuffered by size exclusion chromatography (Sephadex G25 column) and lyophilised in 10 mM HEPES buffer, pH 7.5 (Christ, LT105). 386 mg of lyophilisate were gained with a concentration of 0.15 mg protein/mg lyophilisate according to Bradford double measurements and stored at -20 °C.

Table 4 Purification buffers (pH 7.5) used for Ni-NTA purification and desalting of Δ29*Tf*NCS-A79I by G25-column.

	HEPES [mM], pH 7.5	NaCl [mM]	Imidazole [mM]
Equilibration buffer	100	100	20
Wash buffer	100	100	40
Elution buffer	100	100	500
Desalting buffer	10	0	0

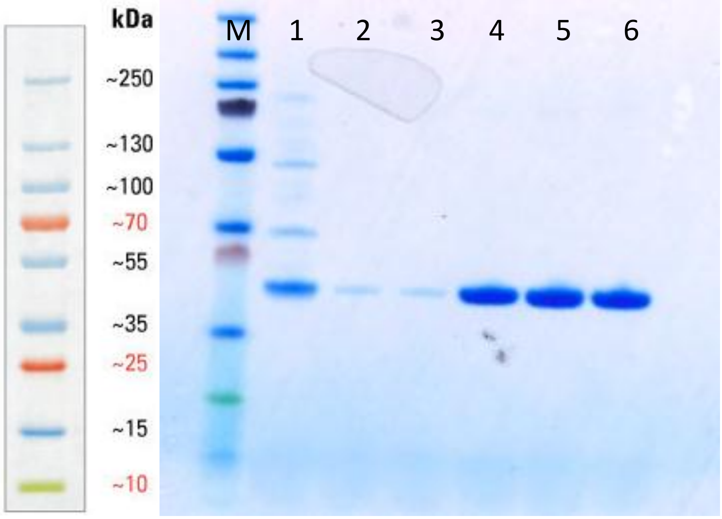

Figure 3 SDS PAGE of Δ29*Tf*NCS-A79I purification. M: protein ladder (PageRuler Plus Prestained, Thermo Fisher Scientific), 1: flow through 1 (at the beginning), 2: flow through 2 (before switching to wash buffer), 3: wash fraction, 4: elution fraction, 5/6: desalting fraction (Δ29*Tf*NCS-A79I after purification).

According to SDS-gel estimation the protein purity was >98% as no further bands were obvious.

1.3 Nucleotide and amino acid sequences of all biocatalysts

1.3.1 *Ec*AHAS-I

Nucleotide sequence (catalytic (CSU: 1758 base pairs) and regulatory subunit (RSU: 291 base pairs))

His-tag underlined, changes compared to AHAS-I wild type are marked grey, 1-1758: catalytic subunit, 1759-2052 regulatory subunit

ATGGGCAGCAGCCATCATCATCATCATCACAGCAGCGGCCTGGTGCCGCGCGGCAGCCATATGGCT
AGCATGGCAAGTTCGGGCACAACATCGACGCGTAAGCGCTTTACCGGCGCAGAATTTATCGTTCATT
TCCTGGAACAGCAGGGCATTAAGATTGTGACAGGCATTCCGGGCGGTTCTATCCTGCCTGTTTACGA
TGCCTTAAGCCAAAGCACGCAAATCCGCCATATTCTGGCCCGTCATGAACAGGGCGCGGGCTTTATC
GCTCAGGGAATGGCGCGCACCGACGGTAAACCGGCGGTCTGTATGGCCTGTAGCGGACCGGGTGC
GACTAACCTGGTGACCGCCATTGCCGATGCGCGGCTGGACTCCATCCCGCTGATTTGCATCACTGGTC
AGGTTCCCGCCTCGATGATCGGCACCGACGCCTTCCAGGAAGTGGACACCTACGGCATCTCTATCCC
CATCACCAAACACAACTATCTGGTCAGACATATCGAAGAACTCCCGCAGGTCATGAGCGATGCCTTCC
GCATTGCGCAATCAGGCCGCCCAGGCCCGGTGTGGATAGACATTCCTAAGGATGTGCAAACGGCAG
TTTTTGAGATTGAAACACAGCCCGCTATGGCAGAAAAAGCCGCCGCCCCCGCCTTTAGCGAAGAAAG
CATTCGTGACGCAGCGGCGATGATTAACGCTGCCAAACGCCCGGTGCTTTATCTGGGCGGCGGTGTG
ATCAATGCGCCCGCACGGGTGCGTGAACTGGCGGAGAAAGCGCAACTGCCTACCACCATGACTTTA
ATGGCGCTGGGCATGTTGCCAAAAGCGCATCCGTTGTCGCTGGGTATGCTGGGGATGCACGGCGTG
CGCAGCACCAACTATATTTTGCAGGAGGCGGATTTGTTGATAGTGCTCGGTGCGCGTTTTGATGACC
GGGCGATTGGCAAAACCGAGCAGTTCTGTCCGAATGCCAAAATCATTCATGTCGATATCGACCGTGC
AGAGCTGGGTAAAATCAAGCAGCCGCACGTGGCGATTCAGGCGGATGTTGATGACGTGCTGGCGCA
GTTGATCCCGCTGGTGGAAGCGCAACCGCGTGCAGAGTGGCACCAGTTGGTAGCGGATTTGCAGCG
TGAGTTTCCGTGTCCAATCCCGAAAGCGTGCGATCCGTTAAGCCATTACGGCCTGATCAACGCCGTTG
CCGCCTGTGTCGATGACAATGCAATTATCACCACCGACGTTGGTCAGCATCAGATGTGGACCGCGCA
AGCTTATCCGCTCAATCGCCCACGCCAGTGGCTGACCTCCGGTGGGCTGGGCACGATGGGTTTTGGC
CTGCCTGCGGCGATTGGCGCTGCGCTGGCGAACCCGGATCGCAAAGTGTTGTGTTTCTCCGGCGACG
GCAGCCTGATGATGAATATTCAGGAGATGGCGACCGCCAGTGAAAATCAGCTGGATGTCAAAATCA
TTCTGATGAACAACGAAGCGCTGGGGCTGGTGCATCAGCAACAGAGTCTGTTCTACGAGCAAGGCG
TTTTTGCCGCCACCTATCCGGGCAAAATCAACTTTATGCAGATTGCCGCCGGATTCGGCCTCGAAACC
TGTGATTTGAATAACGAAGCCGATCCGCAGGCTTCATTGCAGGAAATCATCAATCGCCCTGGCCCGG
CGCTGATCCATGTGCGCATTGATGCCGAAGAAAAAGTTTACCCGATGGTGCCGCCAGGTGCGGCGA
ATACTGAAATGGTGGGGGAA**TAA**GCCATGCAAAACACAACTCATGACAACGTAATTCTGGAGCTCAC
CGTTCGCAACCATCCGGGCGTAATGACCCACGTTTGTGGCCTTTTTGCCCGCCGCGCTTTTAACGTTG
AAGGCATTCTTTGTCTGCCGATTCAGGACAGCGACAAAAGCCATATCTGGCTACTGGTCAATGACGA
CCAGCGTCTGGAGCAGATGATAAGCCAAATCGATAAGCTGGAAGATGTCGTGAAAGTGCAGCGTAA
TCAGTCCGATCCGACGATGTTTAACAAGATCGCGGTGTTTTTCAG**TAA**

Amino acid sequence (CSU: 585 amino acids, 62.9 kDa, RSU: 96 amino acids, 11.1 kDa)

MGSSHHHHHHSSGLVPRGSHMASMASSGTTSTRKRFTGAEFIVHFLEQQGIKIVTGIPGGSILPVYDALS
QSTQIRHILARHEQGAGFIAQGMARTDGKPAVCMACSGPGATNLVTAIADARLDSIPLICITGQVPASMI
GTDAFQEVDTYGISIPITKHNYLVRHIEELPQVMSDAFRIAQSGRPGPVWIDIPKDVQTAVFEIETQPAMA
EKAAAPAFSEESIRDAAAMINAAKRPVLYLGGGVINAPARVRELAEKAQLPTTMTLMALGMLPKAHPLSL
GMLGMHGVRSTNYILQEADLLIVLGARFDDRAIGKTEQFCPNAKIIHVDIDRAELGKIKQPHVAIQADVD
DVLAQLIPLVEAQPRAEWHQLVADLQREFPCPIPKACDPLSHYGLINAVAACVDDNAIITTDVGQHQMW
TAQAYPLNRPRQWLTSGGLGTMGFGLPAAIGAALANPDRKVLCFSGDGSLMMNIQEMATASENQLDV
KIILMNNEALGLVHQQQSLFYEQGVFAATYPGKINFMQIAAGFGLETCDLNNEADPQASLQEIINRPGPA
LIHVRIDAEEKVYPMVPPGAANTEMVGEStop
MQNTTHDNVILELTVRNHPGVMTHVCGLFARRAFNVEGILCLPIQDSDKSHIWLLVNDDQRLEQMISQI
DKLEDVVKVQRNQSDPTMFNKIAVFFQStop

1.3.2 *Cv*2025 transaminase

Nucleotide sequence (1404 base pairs)

His-tag underlined

ATGGGC<u>CATCATCATCATCATCAT</u>ATGCAGAAGCAACGTACGACCAGCCAATGGCGCGAACTGGATG
CCGCCCATCACCTGCATCCGTTCACCGATACCGCATCGCTGAACCAGGCGGGCGCGCGTGATGAC
GCGCGGAGAGGGCGTCTACCTGTGGGATTCGGAAGGCAACAAGATCATCGACGGCATGGCCGGAC
TGTGGTGCGTGAACGTCGGCTACGGCCGCAAGGACTTTGCCGAAGCGGCGCGCCGGCAGATGGAA
GAGCTGCCGTTCTACAACACCTTCTTCAAGACCACCCATCCGGCGGTGGTCGAGCTGTCCAGCCTGCT
GGCTGAAGTGACGCCGGCCGGTTTCGACCGCGTGTTCTATACCAATTCCGGTTCCGAATCGGTGGAC
ACCATGATCCGCATGGTGCGCCGCTACTGGGACGTGCAGGGCAAGCCGGAGAAGAAGACGCTGATC
GGCCGCTGGAACGGCTATCACGGCTCCACCATCGGCGGCGCCAGCCTGGGCGGCATGAAGTACATG
CACGAGCAGGGCGACTTGCCGATTCCGGGCATGGCCCACATCGAGCAGCCTTGGTGGTACAAGCAC
GGCAAGGACATGACGCCGGACGAGTTCGGCGTGGTGGCCGCGCGCTGGCTGGAAGAGAAGATTCT
GGAAATCGGCGCCGACAAGGTGGCCGCCTTCGTCGGCGAACCCATCCAGGGCGCCGGCGGCGTGAT
CGTCCCGCCGGCCACCTACTGGCCGGAAATCGAGCGCATTTGCCGCAAGTACGACGTGCTGCTGGTG
GCCGACGAAGTGATCTGCGGCTTCGGGCGTACCGGCGAATGGTTCGGCCATCAGCATTTCGGCTTCC
AGCCCGACCTGTTCACCGCCGCCAAGGGCCTGTCCTCCGGCTATCTGCCGATAGGCGCGGTCTTTGTC
GGCAAGCGCGTGGCCGAAGGCCTGATCGCCGGCGGCGACTTCAACCACGGCTTCACCTACTCCGGC
CACCCGGTCTGCGCCGCCGTCGCCCACGCCAACGTGGCGGCGCTGCGCGACGAGGGCATCGTCCAG
CGCGTCAAGGACGACATCGGCCCGTACATGCAAAAGCGCTGGCGTGAAACCTTCAGCCGTTTCGAG
CATGTGGACGACGTGCGCGGCGTCGGCATGGTGCAGGCGTTCACCCTGGTGAAGAACAAGGCGAA
GCGCGAGCTGTTCCCCGATTTCGGCGAGATCGGCACGCTGTGCCGCGACATCTTCTTCCGCAACAAC
CTGATCATGCGGGCATGCGGCGACCACATCGTGTCGGCGCCGCCGCTGGTGATGACGCGGGCGGAA
GTGGACGAGATGCTGGCGGTGGCGGAACGCTGTCTGGAGGAATTCGAGCAGACGCTGAAGGCGCG
CGGGCTGGCT**TAG**

Amino acid sequence (467 amino acids, 52.2 kDa)

MGHHHHHHMQKQRTTSQWRELDAAHHLHPFTDTASLNQAGARVMTRGEGVYLWDSEGNKIIDGMA
GLWCVNVGYGRKDFAEAARRQMEELPFYNTFFKTTHPAVVELSSLLAEVTPAGFDRVFYTNSGSESVDT
MIRMVRRYWDVQGKPEKKTLIGRWNGYHGSTIGGASLGGMKYMHEQGDLPIPGMAHIEQPWWYKH
GKDMTPDEFGVVAARWLEEKILEIGADKVAAFVGEPIQGAGGVIVPPATYWPEIERICRKYDVLLVADEVI
CGFGRTGEWFGHQHFGFQPDLFTAAKGLSSGYLPIGAVFVGKRVAEGLIAGGDFNHGFTYSGHPVCAAV
AHANVAALRDEGIVQRVKDDIGPYMQKRWRETFSRFEHVDDVRGVGMVQAFTLVKNKAKRELFPDFG
EIGTLCRDIFFRNNLIMRACGDHIVSAPPLVMTRAEVDEMLAVAERCLEEFEQTLKARGLA

1.3.3 Δ29*Tf*NCS-A79I

Nucleotide sequence (576 base pairs)

His-tag underlined; changes compared to Δ29*Tf*NCS wild type are marked grey

ATGTTGCATCACCAGGGTATCATCAATCAAGTTAGCACCGTCACGAAAGTAATTCATCACGAGCTGG
AAGTTGCGGCATCCGCTGACGACATTTGGACCGTGTACAGCTGGCCGGGTCTGGCGAAGCACTTGCC
GGATCTGCTGCCTGGCATTTTTGAAAAACTGGAGATTATCGGCGATGGCGGTGTTGGTACGATTCTG
GACATGACCTTTGTCCCGGGTGAATTCCCGCACGAGTATAAAGAGAAATTCATCCTGGTTGATAACG
AACATCGTCTGAAGAAGGTGCAGATGATCGAAGGCGGCTATCTGGACCTGGGTGTGACGTATTACA
TGGACACGATTCACGTTGTGCCGACCGGTAAAGACAGCTGCGTCATCAAGAGCAGCACTGAGTACC
ACGTCAAGCCGGAGTTTGTGAAGATTGTTGAGCCGCTGATCACCACCGGTCCACTGGCAGCCATGGC
AGATGCCATTAGCAAGTTGGTCCTGGAACATAAATCTAAAAGCAACTCCGATGAAATTGAGGCGGCG
ATCATCACCGTGCTGGAGCATCACCACCACCATCAC**TGATAA**

Amino acid sequence (190 amino acids, 21.2 kDa)

MLHHQGIINQVSTVTKVIHHELEVAASADDIWTVYSWPGLAKHLPDLLPGIFEKLEIIGDGGVGTILDMTF
VPGEFPHEYKEKFILVDNEHRLKKVQMIEGGYLDLGVTYYMDTIHVVPTGKDSCVIKSSTEYHVKPEFVKIV
EPLITTGPLAAMADAISKLVLEHKSKSNSDEIEAAIITVLEHHHHHH

2. Reaction conditions

2.1 Enzymatic 3-step cascade reaction

Step 1: Carboligation of 3-hydroxybenzaldehyde and sodium pyruvate towards (*R*)-1-hydroxy-1-(3-hydroxyphenyl)propan-2-one

The HEPES-buffer pH 7.5 (100 mM, 238.3 g/mol, Biochrom) was containing 5 mM $MgCl_2$ x 6 H_2O (203.31 g/mol, Fluka Analytical), 0.1 mM thiamine diphosphate (ThDP: 460.77 g/mol, AppliChem), and 0.05 mM flavin adenine dinucleotide disodium salt hydrate (FAD: 829.51 g/mol, Sigma Aldrich), 10 mM 3-hydroxybenzaldehyde (3HBA: 122.12 g/mol, Sigma Aldrich, stored under argon), 20 mM sodium pyruvate (110.04 g/mol, Roth) and 2.5% DMSO

((v/v), 78.13 g/mol, p: 1.1 g/cm^3, Roth) were added. By addition of 0.5 mg/mL purified and lyophilised acetohydroxy acid synthase I from *E. coli* (*Ec*AHAS-I) the reaction was started. The reaction took place in an overall volume of 1 mL, at 30 °C and was stirred with 750 rpm for 1 h (Eppendorf Thermomix) and finally stopped by ultrafiltration of the sample (Merck Millipore Ltd: Microcon-10, centrifugal filters).

Step 2: Transamination of (*R*)-1-hydroxy-1-(3-hydroxyphenyl)propan-2-one and isopropylamine towards metaraminol

900 µL of the step one sample containing the product (*R*)-1-hydroxy-1-(3-hydroxyphenyl)propan-2-one ((*R*)-3HPAC) was diluted to 1200 µL by addition of 3 mg/mL purified and lyophilised transaminase from *Chromobacterium violaceum* (*Cv*2025), 0.2 mM pyridoxal-5-phosphate (PLP, 247.14 g/mol, Sigma Aldrich), 100 mM isopropylamine (IPA: 59.11 g/mol, p: 0.688 g/cm^3, Sigma Aldrich) and 3.1% DMSO (v/v) (78.13 g/mol, p: 1.1 g/cm^3, Roth). The reaction took place in a 1.5 mL glass vial with an overall volume of 1.2 mL at 30 °C and was stirred with 750 rpm with a closed lid. Working with an open lid to possibly shift the reaction equilibrium to the product side was not necessary in the used 1 mL setup. After conversion was completed (8 h), the enzyme was removed by ultrafiltration (Merck Millipore Ltd: Microcon-10, centrifugal filters).

Step 3 (enzymatically catalysed): Cyclisation towards (1*S*,3*S*,4*R*)-1-benzyl-3-methyl-1,2,3,4-tetrahydroisoquinoline-4,6-diol via Pictet-Spengler condensation catalysed by norcoclaurine synthase

925 µL of the second sample was diluted to 1000 µL by addition of 9.5 mM phenylacetaldehyde (120.15 g/mol: 1.075 g/cm^3, 95% purity, Alfa Aesar), 2.5% DMSO (v/v) and 0.5 mg/mL purified and lyophilised ∆29*Tf*NCS-A79I (norcoclaurine synthase variant from *Thalictrum flavum*). The reaction took place in a volume of 1 mL at 37 °C and was stirred with 750 rpm for 3.25 h.

2.2 Chemoenzymatic 3-step cascade reaction

Step 1 and 2 occurred as described above.

Step 3 (chemically catalysed): Cyclisation towards (1*S*,3*S*,4*R*)-1-(2-bromophenyl)-3-methyl-1,2,3,4-tetrahydroisoquinoline-4,6-diol via Pictet-Spengler-condensation catalysed by phosphate buffer

925 µL of the second reaction sample was diluted to 1000 µL by addition of 10 mM 2-bromobenzaldehyde (185.03 g/mol, 98% purity, Sigma Aldrich), and 200 mM potassium phosphate buffer, pH 7.0. The reaction took place at 50 °C and was stirred with 750 rpm for 24 h.

3. Instrumental analysis

3.1 Sample preparation of carboligation measurements (step 1) - HPLC

For conversion and product purity determination of the first carboligation step 20 µL of the sample were diluted in 380 µL acetonitrile, centrifuged for 1 min and measured with HPLC (Agilent LC1260 Infinity, DAD, IE-column from Chiralpak, 5 µm, 4.6 mm x 250 mm, 220 nm, 20 °C, 5 µL injection volume).

Table 5 Gradient method for HPLC IE-column (Chiralpak).

Time [min]	H_2O	Acetonitrile	Flow [mL/min]
0	90	10	0.7
13	90	10	0.7
18	50	50	0.7
18.5	90	10	0.7
21	90	10	1
25	90	10	1

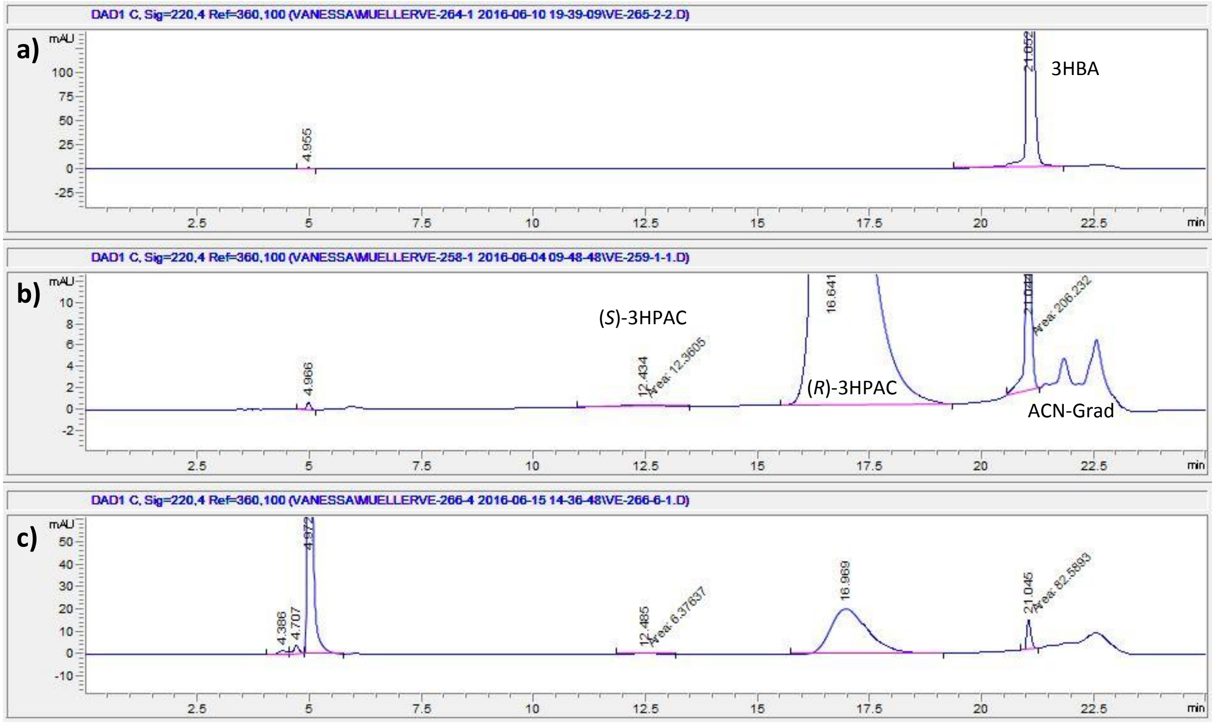

Figure 4 Chromatogram of the measured carboligation from 3HBA and sodium pyruvate to (*R*)-3HPAC after 1 h of reaction time(c), compared to the reference compounds 3HBA (a) and (*R*)- and (*S*)-3HPAC (b).

Table 6 Retention times of the carboligation step compounds measured by HPLC IE-column gradient method (Table 5).

**Substance**	(*S*)-3HPAC	(*R*)-3HPAC	3HBA
**Retention time**	12.4 min	16.9 min	21.0 min

3.2 Sample preparation of transamination measurements (step 2) - HPLC

For conversion measurements 20 µL of the sample were diluted in 180 to 380 µL of acetonitrile + 0.1% DEA, 0.075% TFA and centrifuged for 1 min. The supernatant was measured by HPLC (Agilent LC1260 Infinity, DAD, 100-RP18-column (Lichrospher), 5 µm, 4 mm x 250 mm; 280 nm, 20 °C, 5 µL injection volume).

Table 7 Gradient method for HPLC-100-RP18-column (Lichrospher).

**Time [min]**	**H_2O + 0.075% TFA, 0.1% DEA**	**Acetonitrile + 0.075% TFA, 0.1% DEA**	**Flow [mL/min]**
0	95	5	0.7
11	95	5	0.7
16	50	50	0.75
18	50	50	0.75
18.5	95	5	0.75
23	95	5	0.7

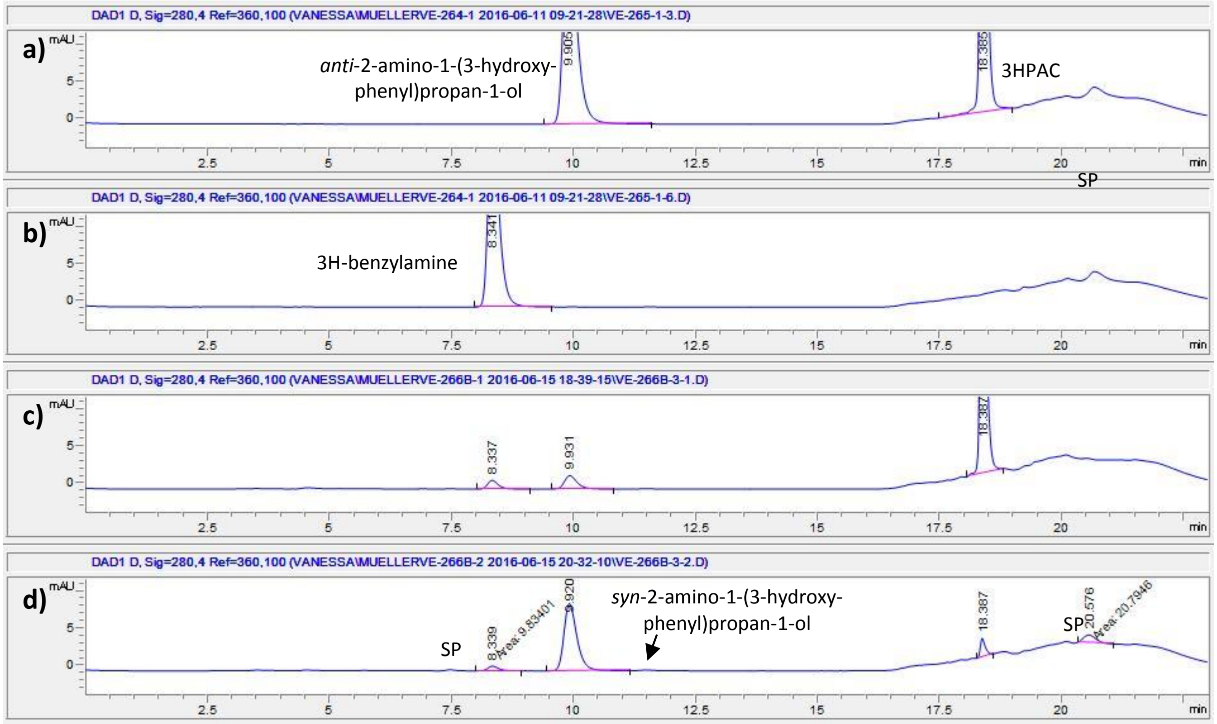

Figure 5 Chromatograms (HPLC) of the transamination reaction from 3HPAC and IPA towards metaraminol after 0 h (c) and 0.5 h (d), compared to the reference compounds 3HPAC and 2-amino-1-(3-hydroxyphenyl)propan-1-ol and the side product 3-hydroxybenzylamine (b).

Table 8 Retention times of the transamination step compounds measured by HPLC-100-RP18-column with gradient method (table 7, SP: unidentified side product).

**Substance**	SP (acetone)	3-hydroxy-benzyl-amine	*anti*-2-amino-1-(3-hydroxy-phenyl) propan-1-ol	*syn*-2-amino-1-(3-hydroxy-phenyl) propan-1-ol	3HPAC	SP
**Retention time (RT)**	7.5 min	8.4 min	9.9 min	11.5 min	18.4 min	20.6 min

3.3 Sample preparation of transamination measurements - SFC

For optical purity measurements of metaraminol, SFC analytics were used (Agilent SFC1260 Infinity, DAD). Two columns were connected in a row to increase the separation efficiency (IA-column (Chiralpak) and AD-H-column (Chiralpak), each 5 µm, 4.6 mm x 250 mm. The mobile phase was a mixture of 88% supercritical CO_2 and 12% MeOH + 0.5% DEA, flow: 0.8 mL/min, 210 nm, 5 µL injection volume, 30 °C.

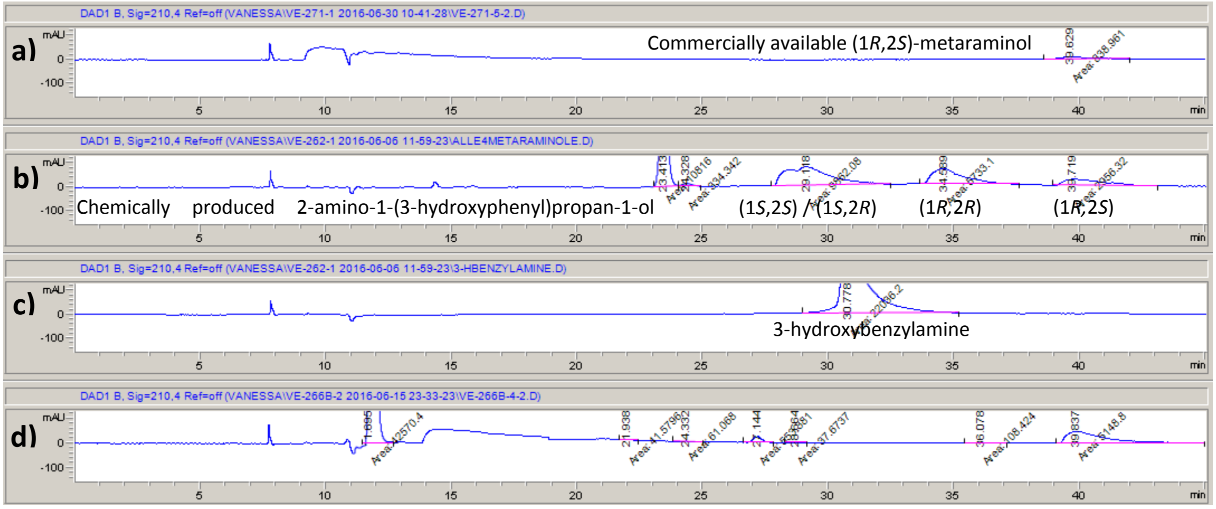

Figure 6 Chromatograms (SFC) of the transamination reaction from 3HPAC and IPA towards metaraminol after 8 h (d), compared to reference compounds of commercially available (1*R*,2*S*)-metaraminol (a), chemically produced isomers of 2-amino-1-(3-hydroxyphenyl)propan-1-ol, identified by enzyme reaction correlation (b) and the side product 3-hydroxybenzylamine (c).

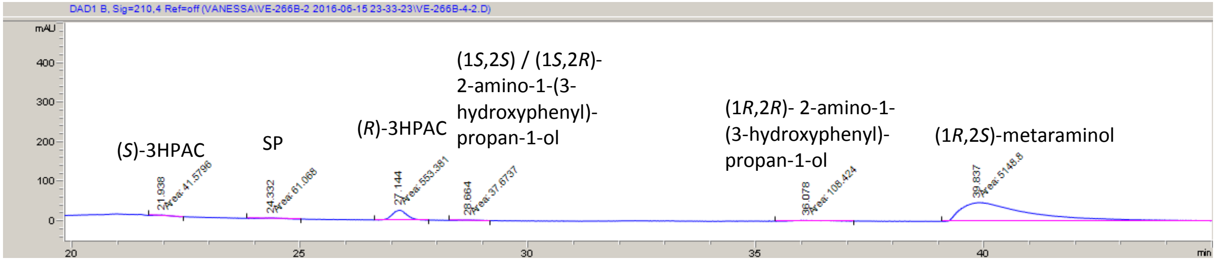

Figure 7 Chromatogram (HPLC) of the transamination reaction from 3HPAC and IPA towards metaraminol after 8 h, zoomed in.

Table 9 Retention times of transamination step compounds measured by SFC (SP: unidentified product).

**Substance**	(*S*)-3HPAC	SP	(*R*)-3HPAC	(1*S*,2*S*)/(1*S*,2*R*)-2-amino-1-(3-hydroxyphenyl) propan-1-ol	(1*R*,2*R*)-2-amino-1-(3-hydroxyphenyl) propan-1-ol	(1*R*,2*S*)-metaraminol
**Retention time (RT)**	22.0 min	24.7 min	27.1 min	28.9 min	36.1 min	40-41 min

3.4 Sample preparation of Pictet-Spengler condensation (step 3) catalysed by NCS - HPLC

For conversion and product purity detection the reaction sample (20 µL) was diluted in 77 – 177 µL MeOH (+ 3 µL 37% HCl), centrifuged for 1 min and the supernatant measured by HPLC with a Supelco column (Astec Chirobiotic T column, 5 µm, 4 mm x 250 mm, 1 mL/min MeOH + 0.2% acetic acid (AcOH) + 0.1% triethylamine (TEA), 25 min, 280 nm, 30 °C, 5 µL injection volume).

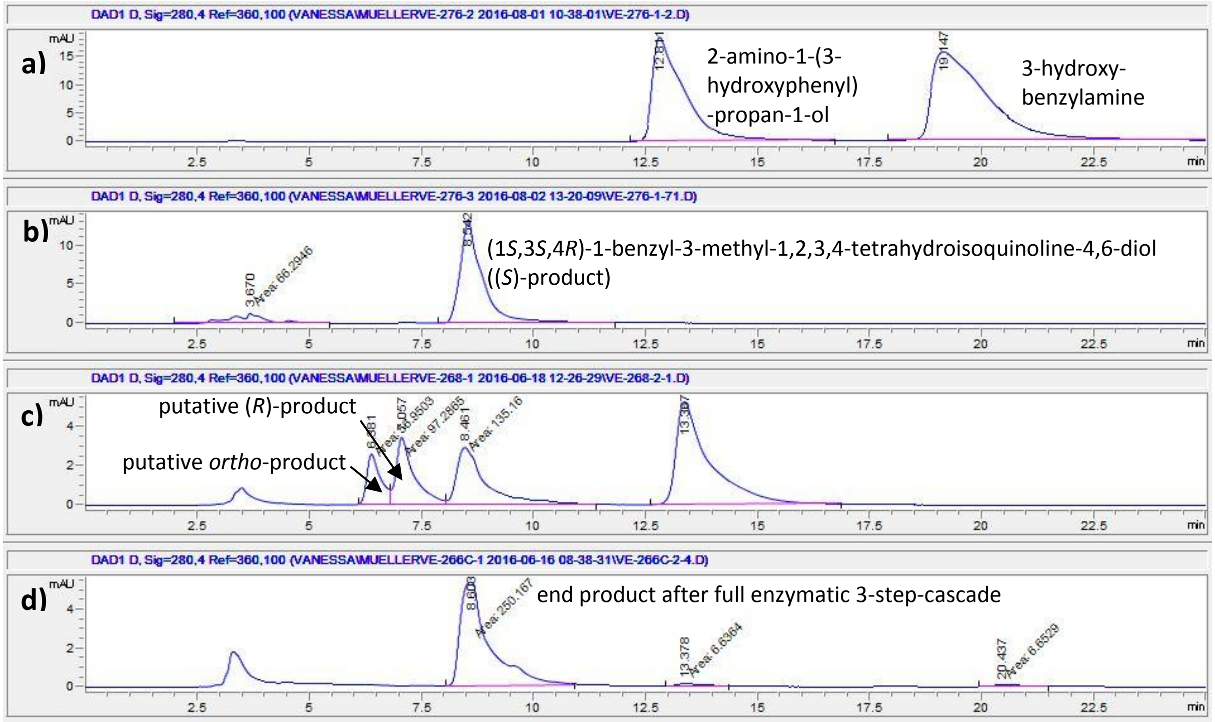

Figure 8 Comparison of the reference compounds (a) 2-amino-1-(3-hydroxyphenyl)propan-1-ol and 3-hydroxybenzylamine, (b) (1*S*,3*S*,4*R*)-1-benzyl-3-methyl-1,2,3,4-tetrahydroisoquinoline-4,6-diol (*S*)-product) to the Pictet-Spengler reaction product (1*S*,3*S*,4*R*)-1-benzyl-3-methyl-1,2,3,4-tetrahydroisoquinoline-4,6-diol ((*S*)-product), catalysed by ∆29*Tf*NCS-A79I (2 h) after full enzymatic 3-step cascade reaction. (c) is the phosphate buffer catalysed Pictet-Spengler reaction from 2-amino-1-(3-hydroxyphenyl)propan-1-ol and phenylacetaldehyde towards 1-benzyl-3-methyl-1,2,3,4-tetrahydroisoquinoline-4,6-diol and 1-benzyl-3-methyl-1,2,3,4-tetrahydroisoquinoline-4,8-diol.[10]

Table 10 Retention times of the Pictet-Spengler condensation compounds, measured by HPLC Supelco-column. (*S*)-isoquinoline: (1*S*,3*S*,4*R*)-1-benzyl-3-methyl-1,2,3,4-tetrahydroisoquinoline-4,6-diol, (*R*)-isoquinoline: (1*R*,3*S*,4*R*)-1-benzyl-3-methyl-1,2,3,4-tetrahydroisoquinoline-4,6-diol.

**Substance**	Putative *ortho* product	Putative (*R*)-isoquinoline	(*S*)-isoquinoline	2-amino-1-(3-hydroxyphenyl) propan-1-ol	3-H-benzylamine
**Retention times (RT)**	6.4 min	7.1 min	8.5 min	13.4 min	20.4 min

3.5 Sample preparation of Pictet-Spengler reaction catalysed by potassium phosphate - HPLC

The reaction sample (20 µL) was diluted in 77 – 177 µL MeOH (+ 3 µL 37% HCl), centrifuged for 1 min and the supernatant measured by HPLC with a Supelco column (Astec Chirobiotic T column, 5 µm, 4 mm x 250 mm, 0.8 mL/min MeOH + 0.2% acetic acid (AcOH) + 0.1% triethylamine (TEA), 30 min, 280 nm, 30 °C, 5 µL injection volume).

The products were identified by comparison with the reference compound (Fig. 9) and detection by GC-TOF-MS (see Fig. 10-15). Conversions were calculated by substrate depletion of 2-amino-1-(3-hydroxyphenyl)propan-1-ol.

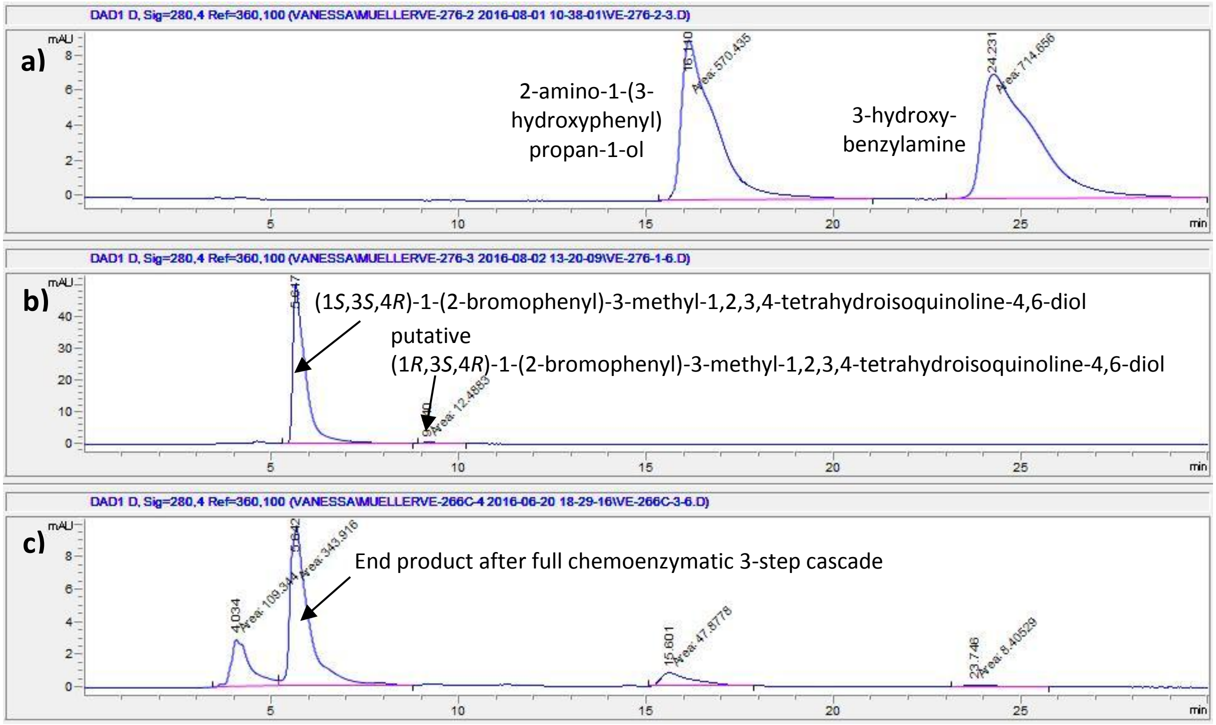

Figure 9 Chromatograms of the Pictet-Spengler reaction from 2-amino-1-(3-hydroxyphenyl)propan-1-ol and 2-bromobenzaldehyde towards (1*S*,3*S*,4*R*)-1-(2-bromophenyl)-3-methyl-1,2,3,4-tetrahydroisoquinoline-4,6-diol catalysed by potassium phosphate (24 h) after full chemoenzymatic 3-step cascade reaction (c), compared to the reference compounds 2-amino-1-(3-hydroxyphenyl)propan-1-ol and 3-hydroxybenzylamine (a) and (1*S*,3*S*,4*R*)-1-(2-bromophenyl)-3-methyl-1,2,3,4-tetrahydroisoquinoline-4,6-diol (b).

Table 11 Retention times of the Pictet-Spengler condensation compounds, measured with an HPLC Supelco-column. (*S*)-isoquinoline: (1*S*,3*S*,4*R*)-1-(2-bromophenyl)-3-methyl-1,2,3,4-tetrahydroisoquinoline-4,6-diol, (*R*)-isoquinoline: (1*R*,3*S*,4*R*)-1-(2-bromophenyl)-3-methyl-1,2,3,4-tetrahydroisoquinoline-4,6-diol.

**Substance**	(*S*)-isoquinoline	(*R*)-isoquinoline	2-amino-1-(3-hydroxyphenyl)-propan-1-ol	3H-benzylamine
**Retention times (RT)**	5.6 min	9.2 min	15.6 min	23.8 min

3.6 Product identification by GC-TOF-MS

Sample preparation (lyophilisation and derivatisation) and GC-TOF-MS measurements were kindly provided by Jochem Gätgens, Bioprocess & Bioanalytics group, IBG-1: Biotechnology, Forschungszentrum Jülich GmbH, according to a protocol described elsewhere.[11]

3.6.1 Sample preparation and derivatisation

The samples (130 µL) were shock frozen in liquid nitrogen, lyophilised overnight (Christ LT-105 freeze drier (Martin Christ Gefriertrocknungsanlagen, Osterode am Harz, Germany) and stored at -20 °C.

Dried samples were consecutively derivatised with 50 µL of 20 mg/mL O-methylhydroxylamine (MeOX) in pyridine for 90 min at 30 °C and 600 rpm in an Eppendorf Thermomixer followed by an incubation with additional 80 µL of N-acetyl-N-(trimethylsilyl)-trifuoroacetamide (MSTFA) for 90 min at 40 °C and 600 rpm.

For the determination of the derivatised metabolites an Agilent 6890N gas chromatograph (Agilent, Waldbronn, Germany) was used coupled to a Waters Micromass GCT Premier high resolution time of flight mass spectrometer (Waters, Eschborn, Germany). The system was controlled by Waters MassLynx 4.1 software. Injections were performed by a Gerstel MPS 2 (Gerstel, Mülheim ad Ruhr, Germany) controlled by Maestro software.[11]

3.6.2 MS Data Acquisition

1 µL sample was injected into a split/splitless injector at 280 °C at varying split modes. The GC was equipped with a 30 m Agilent EZ-Guard VF-5ms + 10 m guard column (Agilent, Waldbronn, Germany). Constant helium flow was set to 1 mL/min. The GC temperature program started at 60 °C with a hold time of 2 min, followed by a temperature increase of +12 °C/min up to the final temperature of 300 °C, hold time 8 min (total run time of 30 min). The transferline temperature was set to 300 °C. The ToF MS was operated in positive electron impact $[EI]^+$ mode at an electron energy of 70 eV. Ion source temperature was set to 180 °C. The MS was tuned and calibrated with the mass fragmentation pattern of heptacosafluoro-tributylamine (Heptacosa). During analysis the accurate masses were corrected to a single point lockmass of chloro-pentafluoro-benzene (CPFB) as an external reference at 201.9609 m/z. Data acquisition was done in centroid mode with a scanrate of 0.09 s and an interscan delay of 0.01 s that means 10 scans/sec.[11]

3.6.3 Peak identification

For the identification of known metabolites we used a baseline noise subtracted fragment pattern in comparison to our inhouse database JuPoD, the commercial database NIST11 (National Institute of Standards and Technology, USA) and the free available database GMD (MPI of Molecular Plant Physiology, Golm, Germany).[11],[12]

Unknown peaks were identified by structural combination of elemental compositions and verified by virtual derivatisation and fragmentation of the predicted structures.

3.6.4 GC-TOF-MS data of the 3-step cascade reaction.

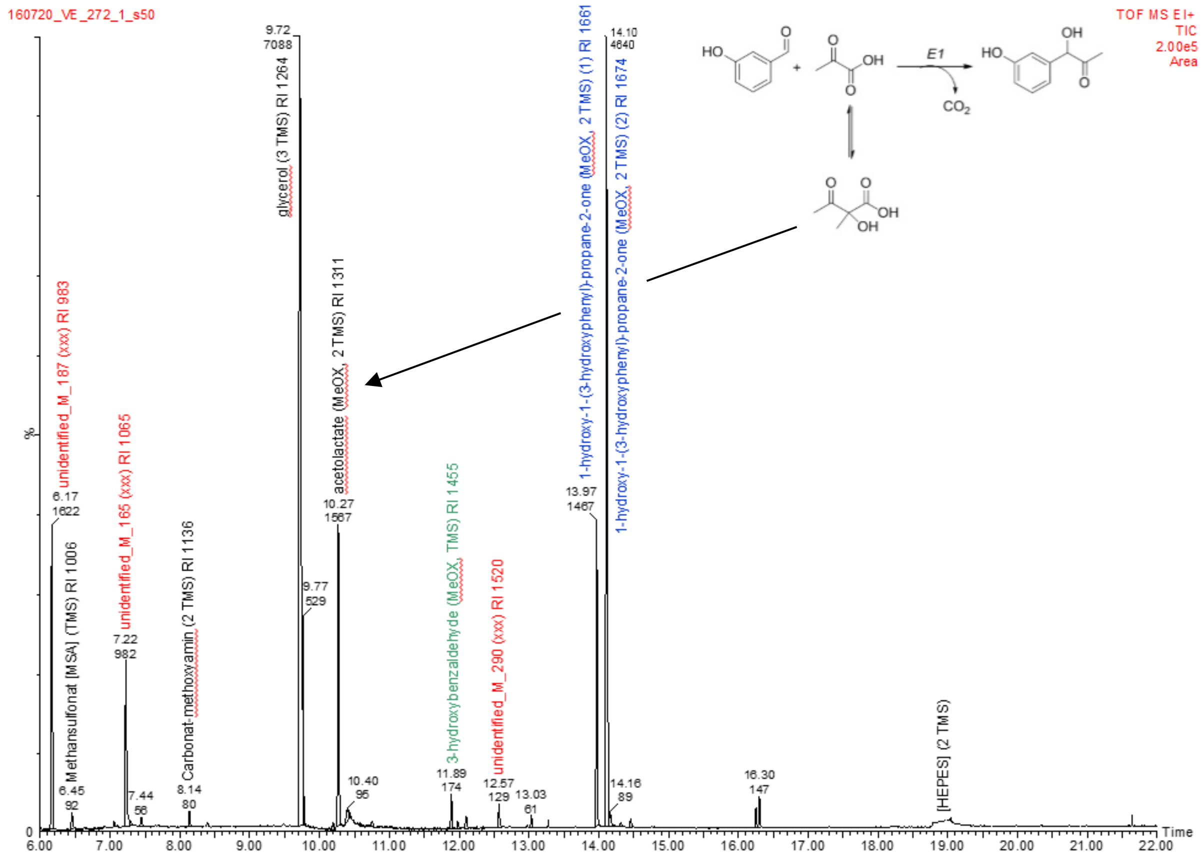

Figure 10 GC-TOF-MS data of the carboligation step (1-hydroxy-1-(3-hydroxyphenyl)propan-2-one is identified as main product of this step, glycerol is found in the reaction mixture, due to the use of not purged syringe filters).

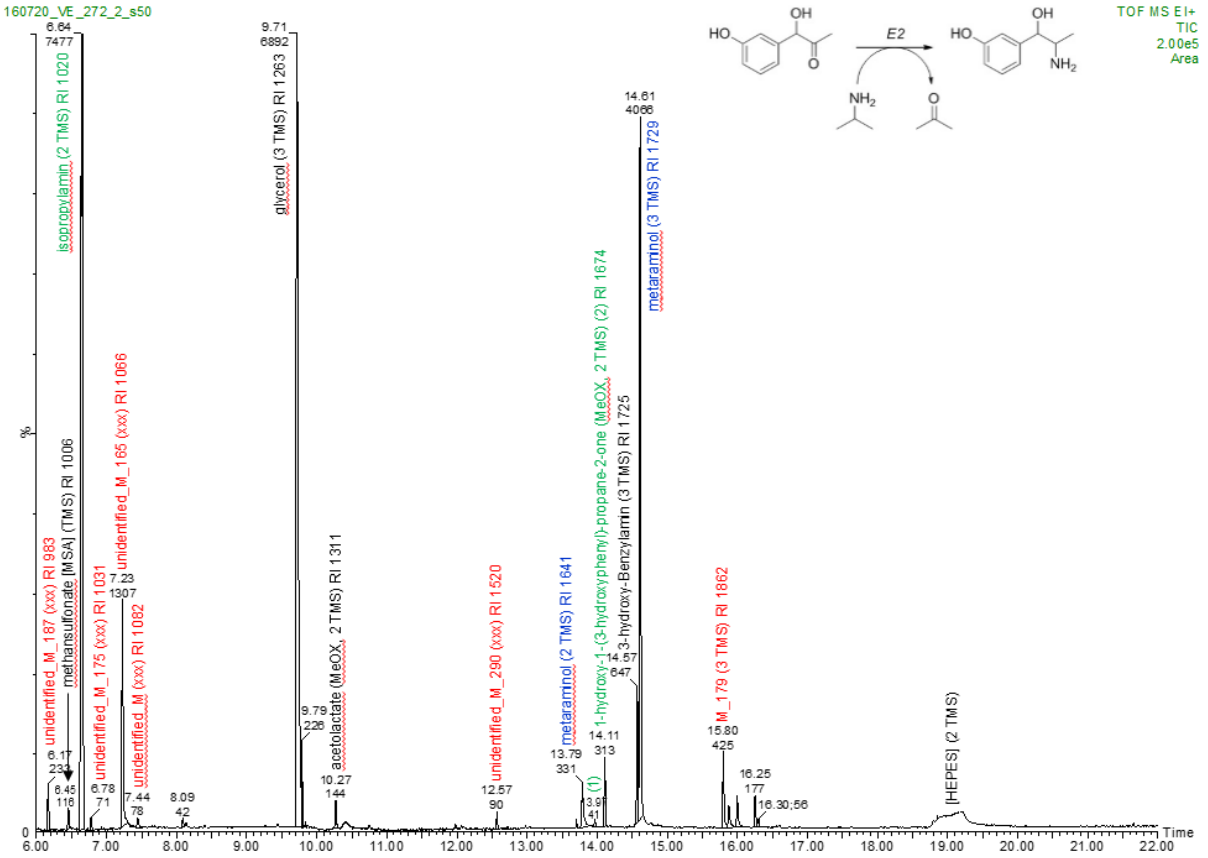

Figure 11 GC-TOF-MS data of the transamination step, 2-amino-1-(3-hydroxyphenyl)propan-1-ol is identified as main product of this step, glycerol is found in the reaction mixture, due to the use of not purged syringe filters.

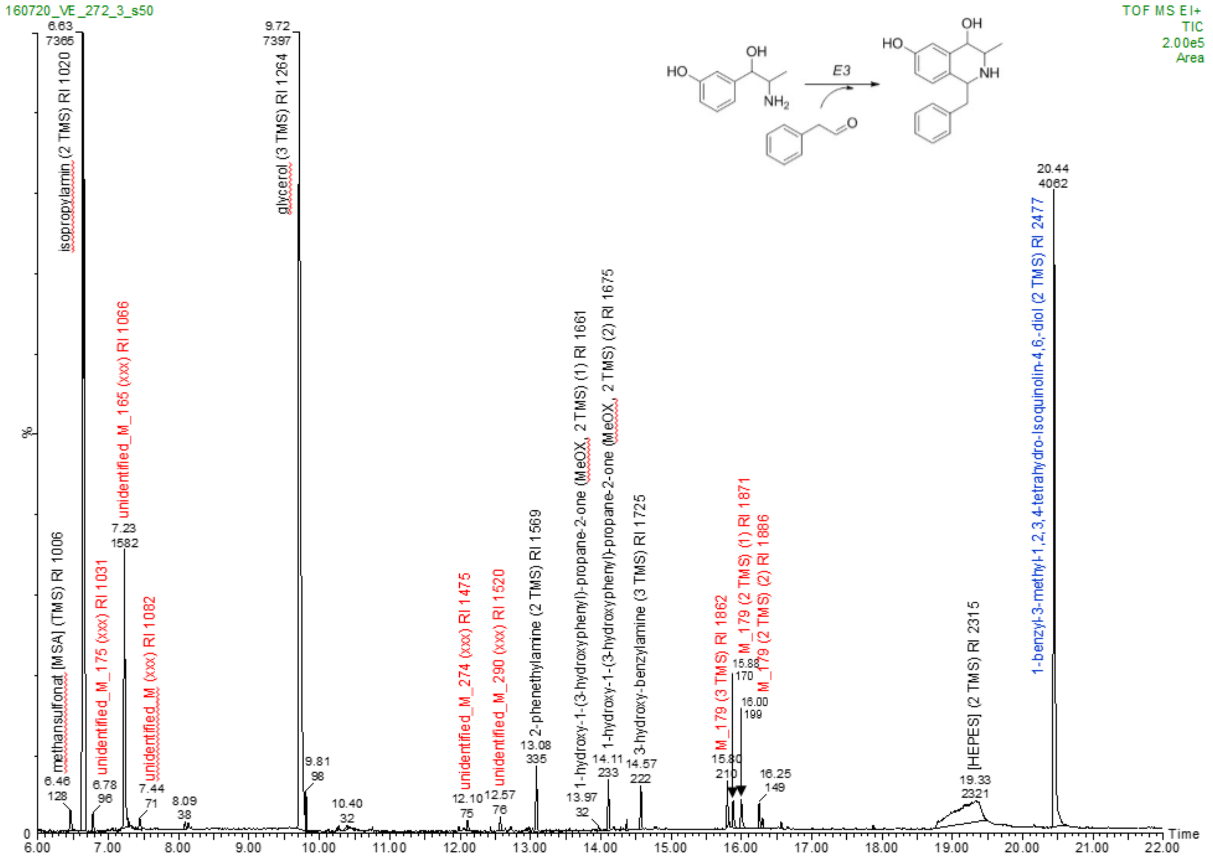

Figure 12 GC-TOF-MS data of the Pictet-Spengler condensation step (1-benzyl-3-methyl-1,2,3,4-tetrahydroisoquinoline-4,6-diol is identified as main product of this step, glycerol is found in the reaction mixture due to the use of not purged syringe filters).

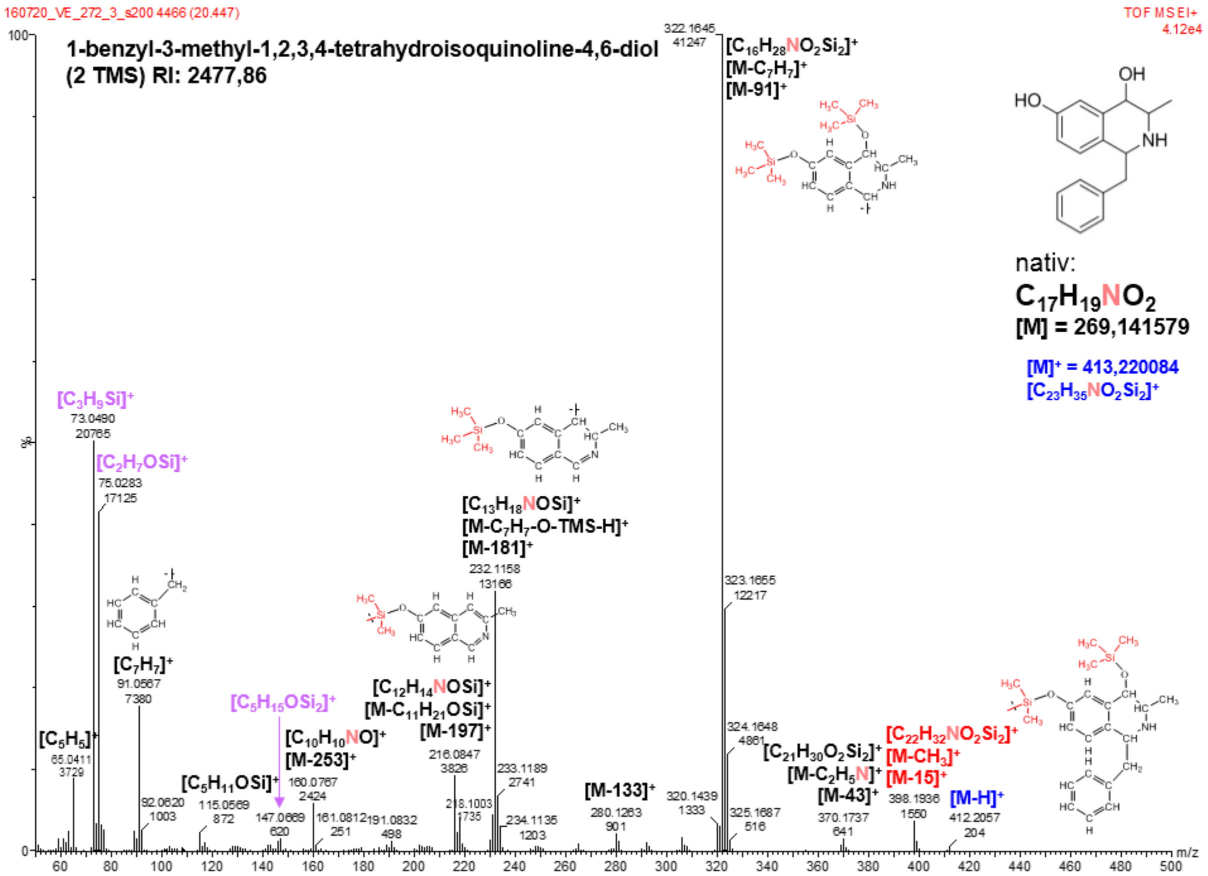

Figure 13 Fragmentation pattern of the 3-step cascade end product 1-benzyl-3-methyl-1,2,3,4-tetrahydroisoquinoline-4,6-diol (2 TMS derivatisation).

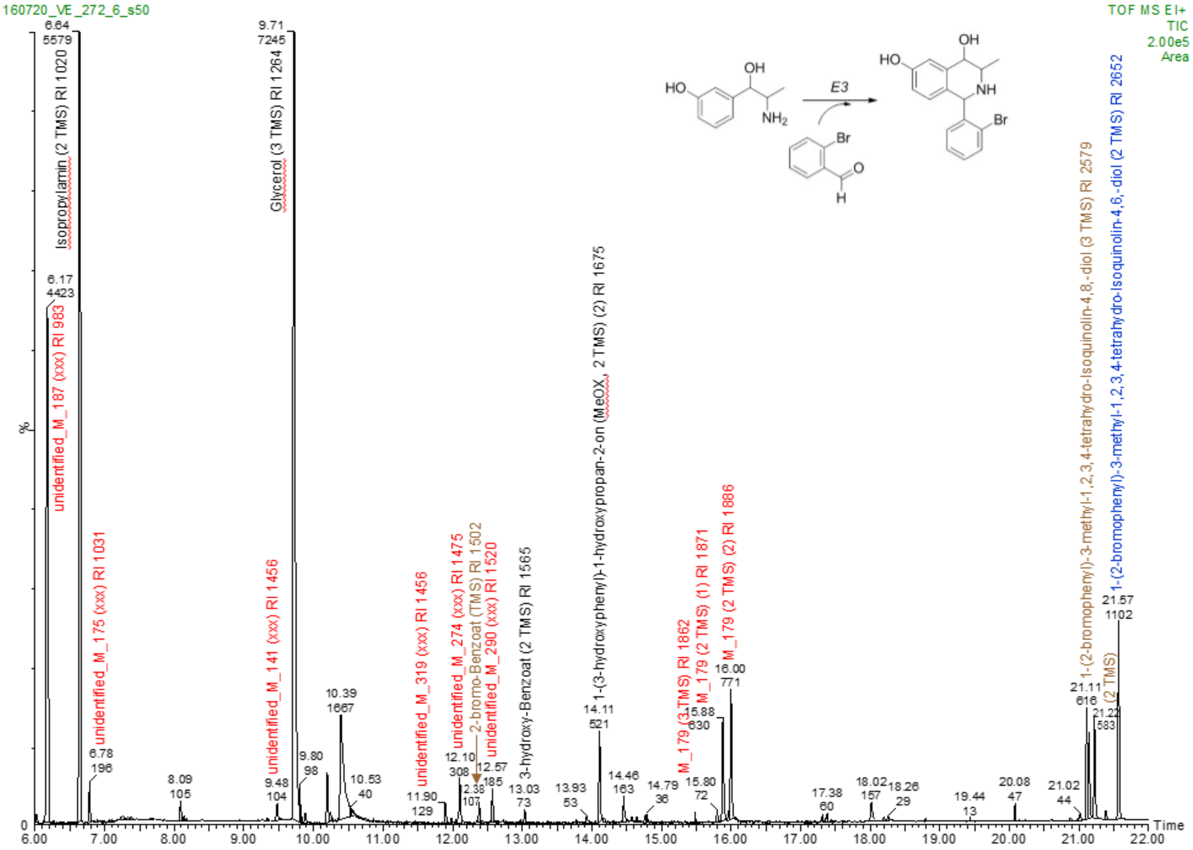

Figure 14 GC-TOF-MS data of the Pictet-Spengler condensation step in which 1-(2-bromophenyl)-3-methyl-1,2,3,4-tetrahydroisoquinoline-4,6-diol is identified as main product of this step. Furthermore, the putative *ortho*-product 1-(2-bromophenyl)-3-methyl-1,2,3,4-tetrahydroisoquinoline-4,8-diol is found as a minor product, glycerol is found in the reaction mixture due to the use of not purged syringe filters.

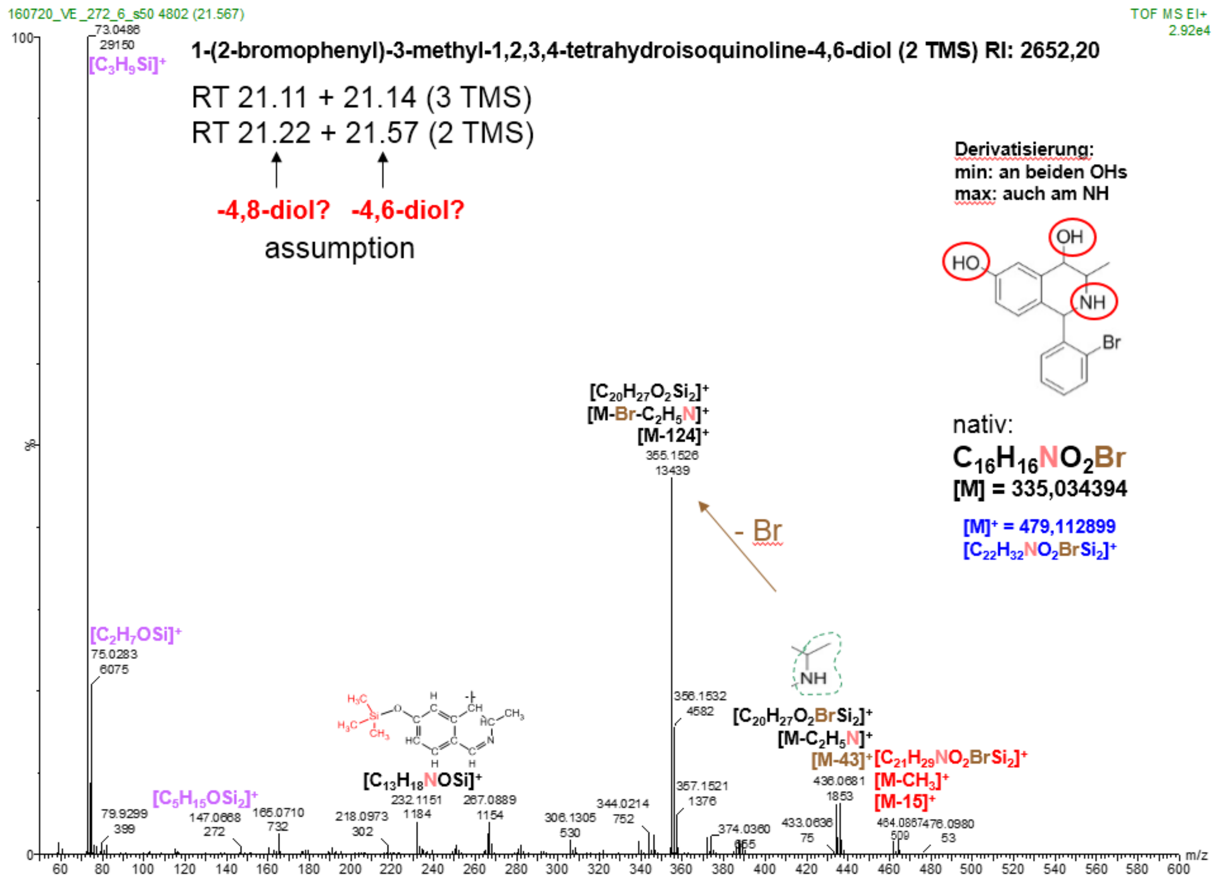

Figure 15 Fragmentation pattern of the 3-step cascade end product 1-(2-bromophenyl)-3-methyl-1,2,3,4-tetrahydroisoquinoline-4,6-diol (2 TMS derivatisation).

4. Synthesis of reference compounds

4.1 (1*S*,3*S*,4*R*)-1-benzyl-3-methyl-1,2,3,4-tetrahydroisoquinoline-4,6-diol synthesis

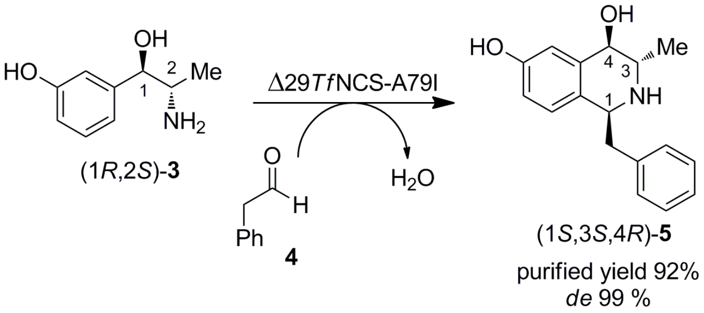

Scheme 1 Reference compound synthesis.

To (1*R*,2*S*)-metaraminal bitartrate (15.9 mg, 0.050 mmol; 10 mM) and sodium ascorbate (4.5 mg, 0.023 mmol; 5 mM), in HEPES buffer (pH 7.5, 3.5 mL; 50 mM) and DMSO (500 µL), phenylacetaldehyde (8.5 µL, 0.076 mmol; 15 mM) was added dropwise, afterwards the purified catalyst Δ29*Tf*NCS-A79I (0.5 mg/mL (in 100 mM HEPES buffer pH 7.5), 1.04 mL) was added. The mixture was stirred under argon at 37 ^{o}C for 18 h. A solution of 1 M HCl (0.5 mL) was added and the mixture centrifuged (4000 rpm for 10 min). The supernatant was diluted two times using distilled water (5.5 mL), adjusted pH to 7.5 (1 M NaOH) and extracted with ethyl acetate (3 × 10 mL). The ethyl acetate extracts were subsequently washed with water (3 × 10 mL), dried (Na_2SO_4) and the solvent removed *in vacuo*. The residue was resuspended in 1 M HCl (5 mL), and extracted with dimethyl carbonate (4 × 5 mL). The solvent was evaporated (including co-evaporation with methanol) to obtain the product HCl salt as a colorless solid (14 mg, 92%; 99.4% purity). The solid was further washed with methanol (0.5 mL) to give the titled compound as colorless crystals (8 mg, 53%; >99.8% purity). Mp 220–223 °C (methanol); $[\alpha]^{20}_{D}$ 35.1 (*c* 0.5, MeOH); ^{1}H NMR (600 MHz; CD_3OD) δ 7.34–7.39 (m, 2H, 2 x Ph 3-H), 7.32 (t, *J* = 7.3 Hz, 1H, Ph 4-H), 7.27 (d, *J* = 7.1 Hz, 2H, 2 x Ph 2-H), 6.96 (d, *J* = 2.4 Hz, 1H, 5-H), 6.72 (d, *J* = 8.5 Hz, 1H, 8-H), 6.66 (dd, *J* = 8.5, 2.5 Hz, 1H, 7-H), 4.71 (t, *J* = 7.0 Hz, 1H, 1-H), 4.51 (d, *J* = 6.8 Hz, 1H, 4-H), 3.62 (quint, *J* = 6.8 Hz, 1H, 3-H), 3.32 (m, 2H, CH_2Ph), 1.39 (d, *J* = 6.8 Hz, 3H, CH_3); ^{13}C NMR (151 MHz; CD_3OD) δ 159.0, 137.0, 136.7, 130.9, 130.1, 129.2, 128.7, 122.8, 116.7, 115.9, 70.2, 56.4, 53.0, 49.9, 41.7, 15.3; *m/z* [ES+] 270 ([MH]$^{+}$, 100%), 252 (21); *m/z* [HRMS ES+] found [MH]$^{+}$ 270.1487. $C_{17}H_{20}NO_2$ requires 270.1489.

Chiral HPLC was conducted on an Agilent Technologies 1260 Infinity machine with a UV detector (at 280 nm), using an Astec Chirobiotic™ T column (25 cm × 4.6 mm, 5 µm) at 25 °C. The product (injection volume 5 µL) was eluted with MeOH (containing 0.2% AcOH and 0.1% TEA) at 1 mL/min and the (1*S*,3*S*,4*R*)-product had a retention time (r_t) of 8.5 min.

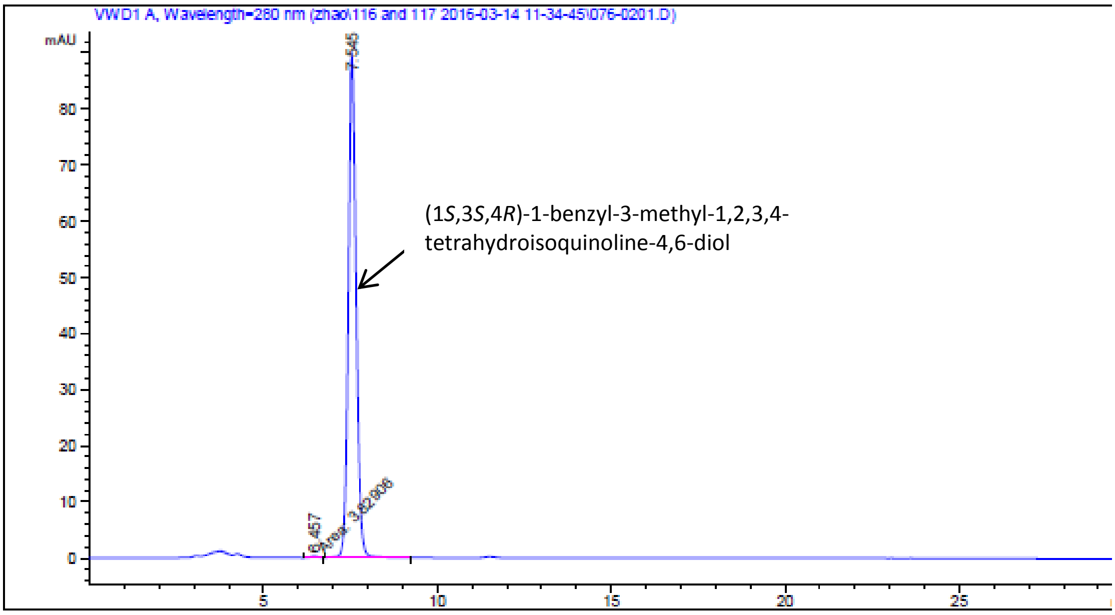

Figure 16 (1*S*,3*S*,4*R*)-1-benzyl-3-methyl-1,2,3,4-tetrahydroisoquinoline-4,6-diol (*de* 99.4%) with HPLC Astec Chirobiotic™ T column.

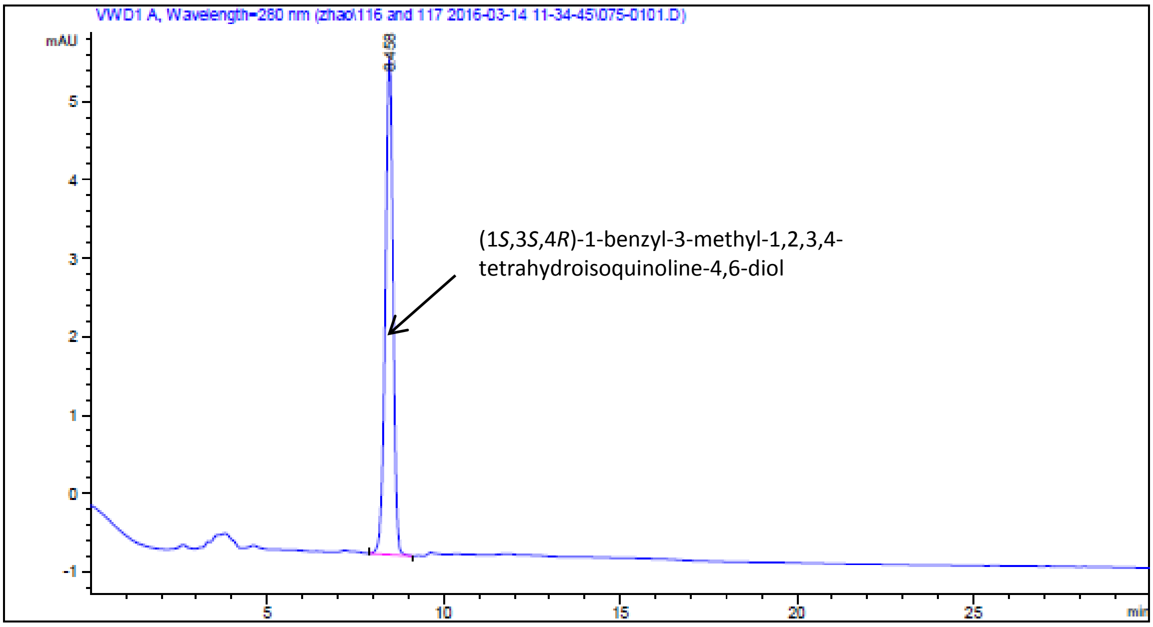

Figure 17 (1*S*,3*S*,4*R*)-1-benzyl-3-methyl-1,2,3,4-tetrahydroisoquinoline-4,6-diol (*de* >99.8%) with HPLC Astec Chirobiotic™ T column.

NMR data

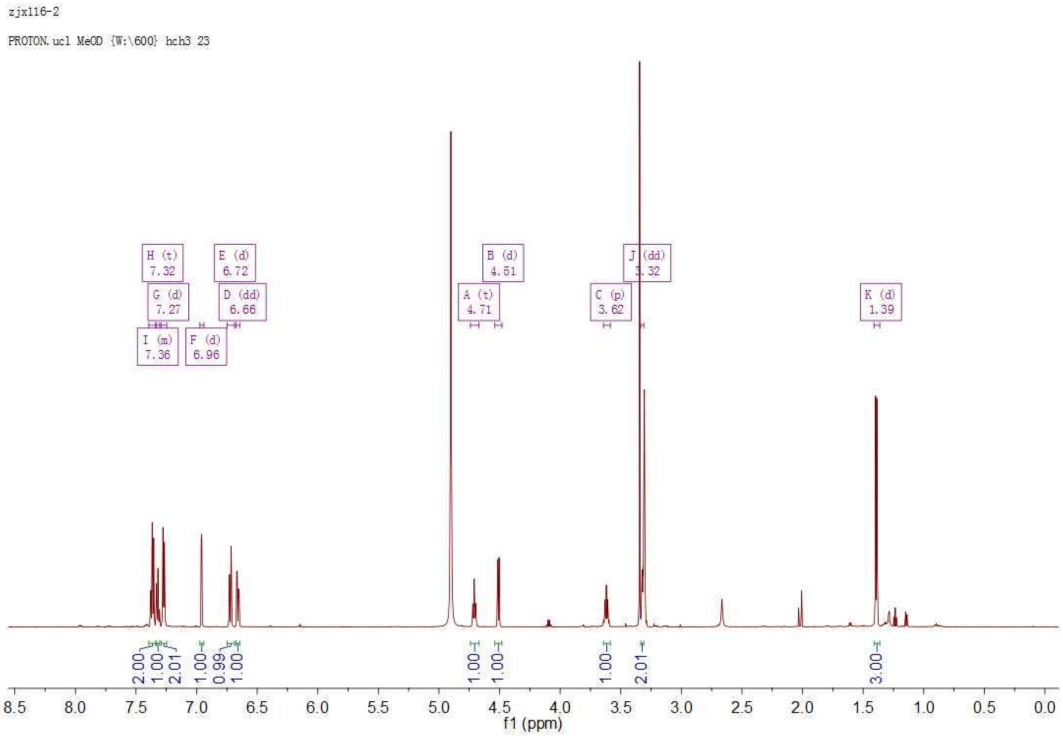

Figure 18 ^{1}H NMR.

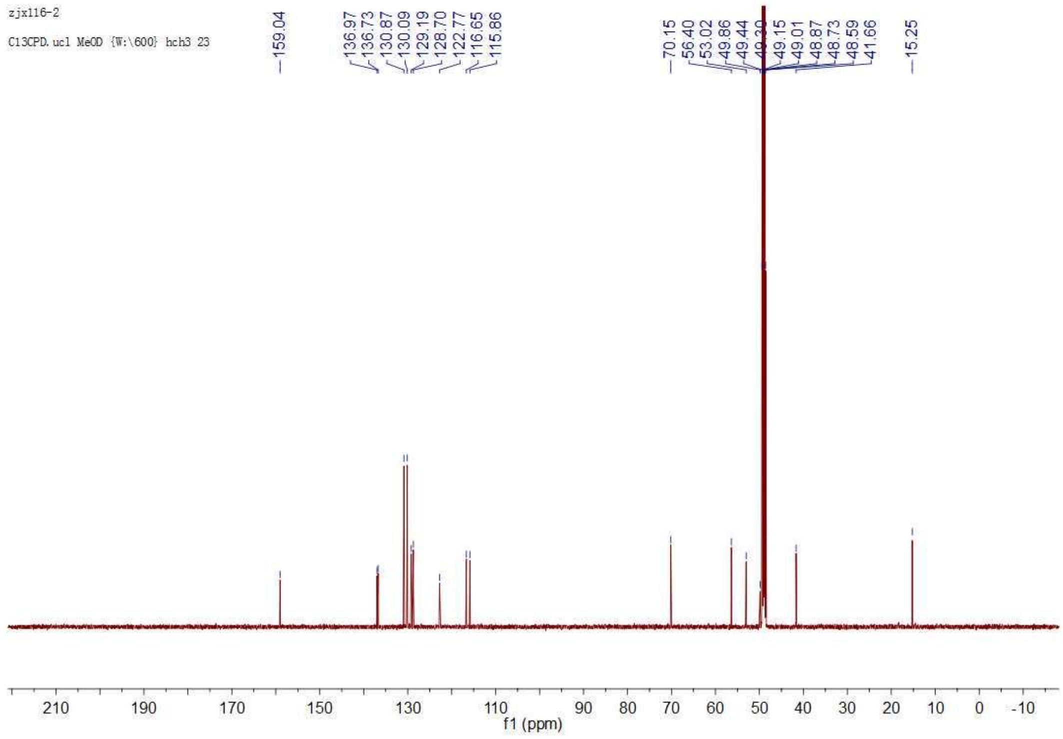

Figure 19 ^{13}C NMR.

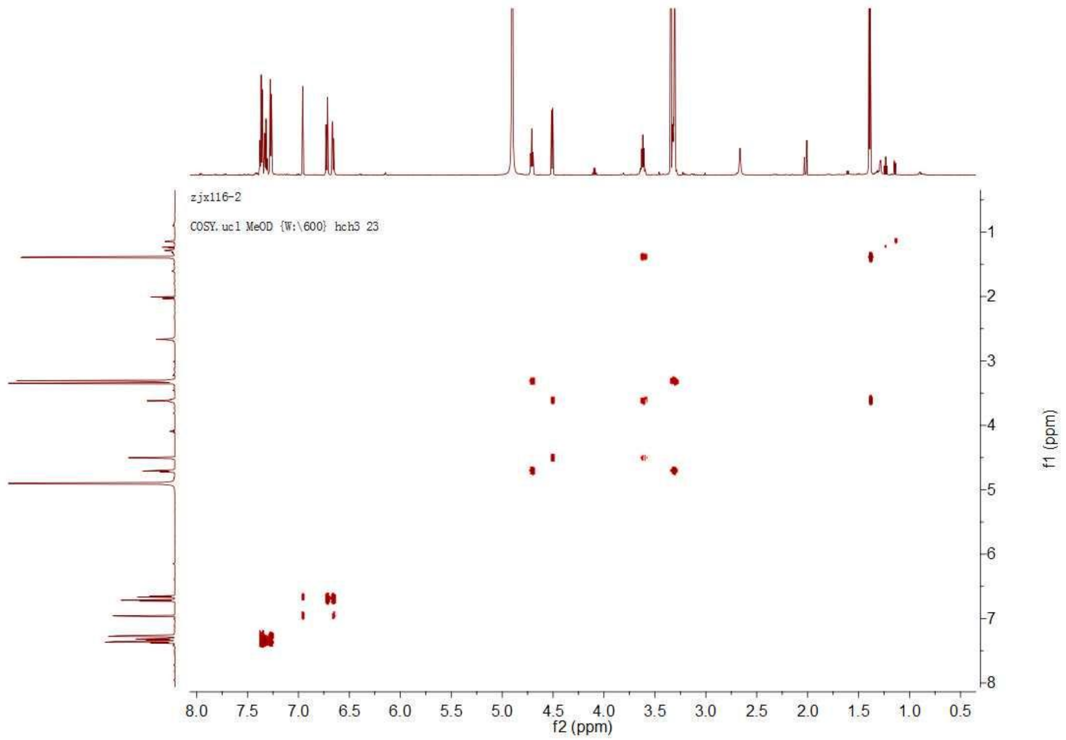

Figure 20 ^{1}H-^{1}H COSY.

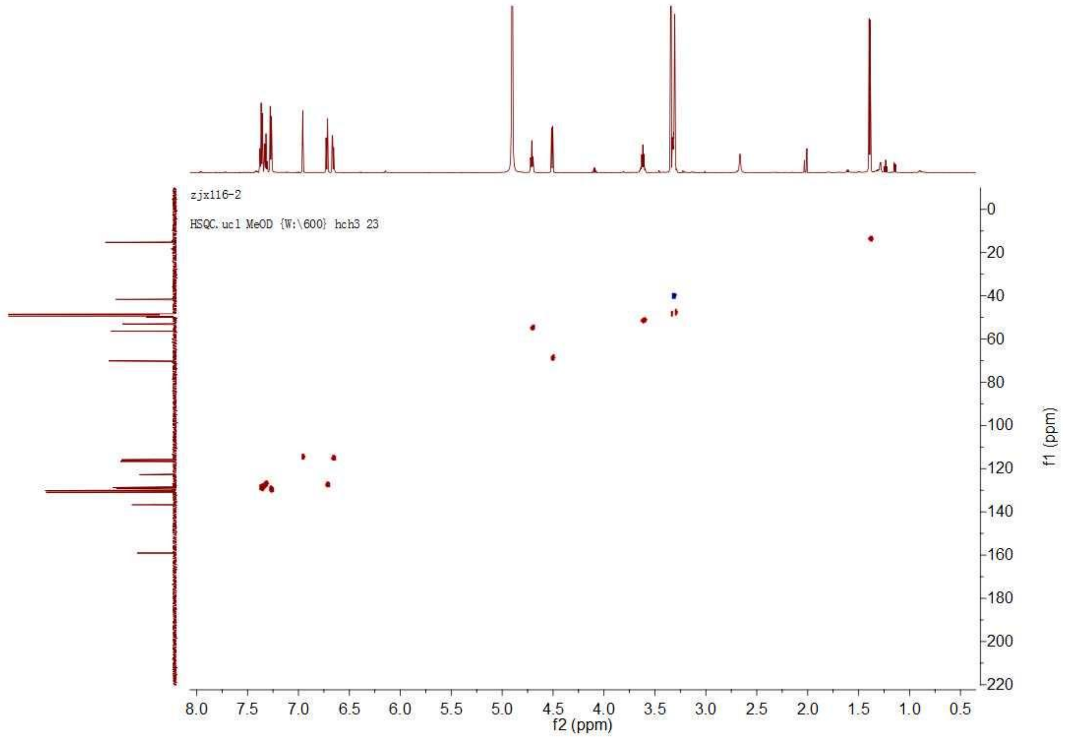

Figure 21 HSQC.

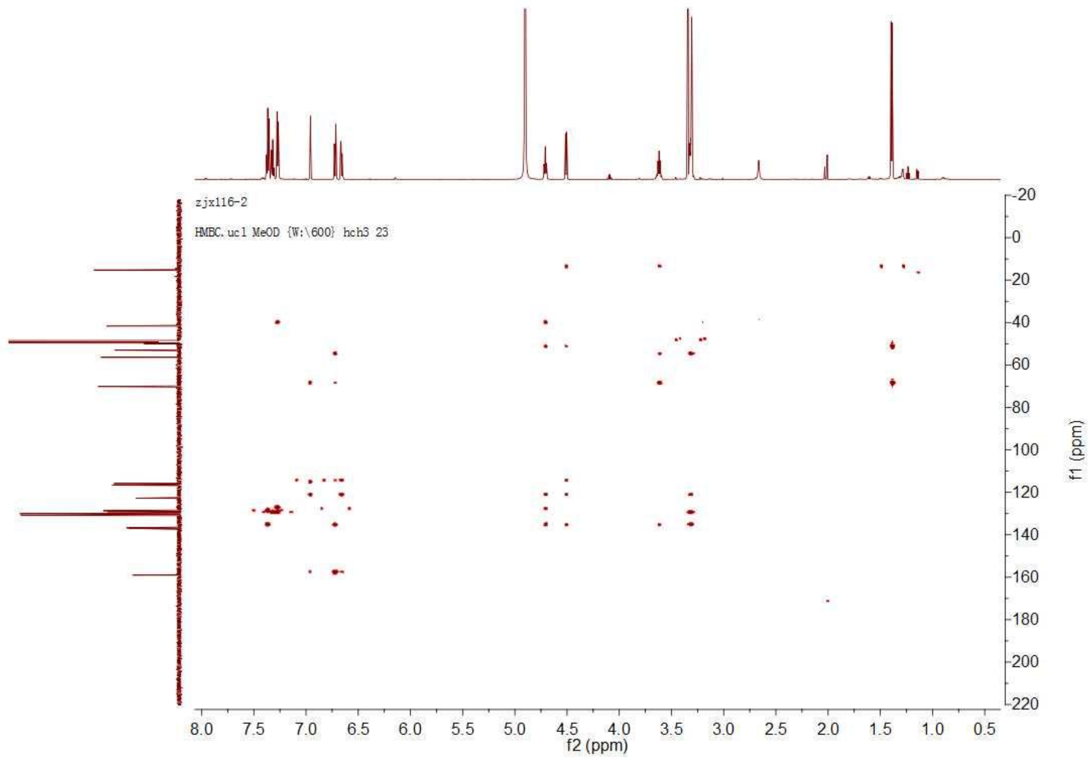

Figure 22 HMBC.

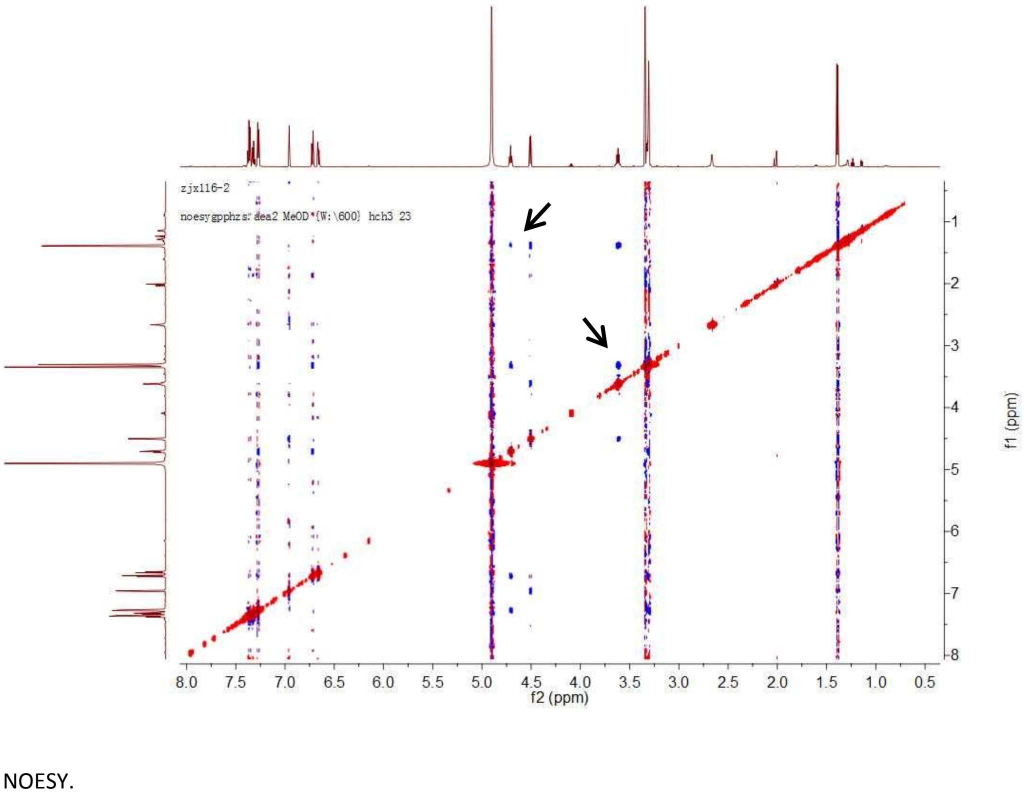

Figure 23 NOESY.

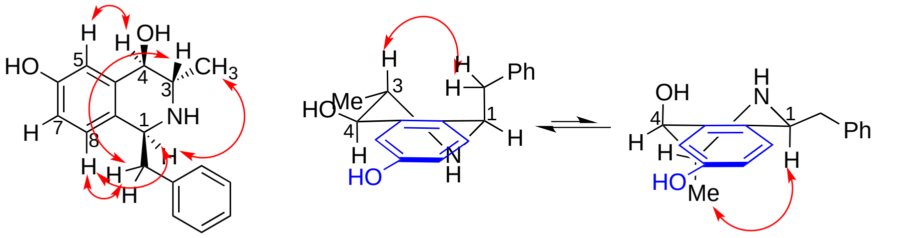

Scheme 2 Key NOEs were observed between 1-H and 3-CH_3, and also between the 1-CH_2 group and 3-H, confirming that the stereochemistry at C-1 is the (*S*)-configuration.

4.2 (1*S*,3*S*,4*R*)-1-(2-bromophenyl)-3-methyl-1,2,3,4-tetrahydroisoquinoline-4,6-diol synthesis

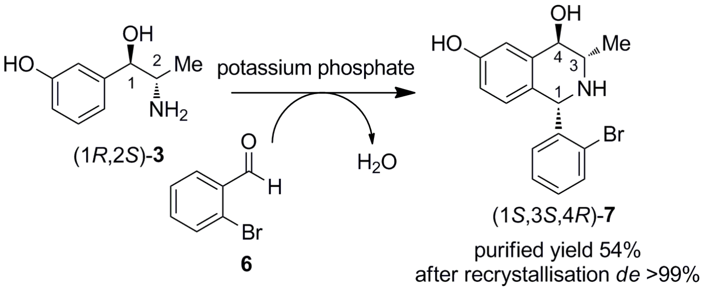

Scheme 3 Reference compound synthesis.

To (1*R*,2*S*)-metaraminal bitartrate (63.5 mg, 0.20 mmol; 20 mM) and sodium ascorbate (18.0 mg, 0.091 mmol; 10 mM), in potassium phosphate buffer (pH 7.0, 9 mL; 1 M) and DMSO (1 mL), 2-bromobenzaldehyde (55.5 mg, 0.30 mmol; 30 mM) was added. The reaction was stirred under argon at 60 °C for 18 h. The solution was cooled to RT, adjusted to pH 7.5 using 1 M NaOH, then extracted with ethyl acetate (3 × 15 mL), dried (Na_2SO_4) and the solvent removed *in vacuo*. The residue was resuspended in 1 M HCl (5 mL), and extracted with dimethyl carbonate (4 × 5 mL). The solvent was evaporated (including co-evaporation with methanol) to obtain the product HCl salt as a colorless solid (40 mg, 54%; 97% purity). The product was further purified by recrystallisation (1:2 ethanol/diethyl ether) and washed with cold ethanol (2 × 1 mL) to give the titled compound as colorless crystals (17 mg, 23%; >99% purity). Mp 212–214 °C (ethanol/diethyl ether); $[\alpha]^{20}_D$ -13.9 (*c* 0.25, MeOH); ^{1}H NMR (600 MHz; CD_3OD) δ 7.79–7.84 (m, 1H), 7.48–7.51 (m, 1H), 7.40–7.44 (m, 2H), 7.12 (dd, *J* = 2.6, 0.7 Hz, 1H, 5-H), 6.66 (dd, *J* = 8.6, 2.6 Hz, 1H, 7-H), 6.40 (dd, *J* = 8.6, 0.8 Hz, 1H, 8-H), 6.16 (s, 1H, 1-H), 4.81 (d, *J* = 9.6 Hz, 1H, 4-H), 3.65 (dq, *J* = 9.6, 6.5 Hz, 1H, 3-H), 1.61 (d, *J* = 6.5 Hz, 3H, CH_3); ^{13}C NMR (151 MHz; CD_3OD) δ 159.5, 139.6, 137.3, 134.8, 132.8, 131.4, 130.1, 129.2,

126.5, 123.0, 116.9, 114.8, 70.5, 61.9, 58.4, 16.2; *m/z* [ES+] 336 (95%, $[MH]^+$ (^{81}Br)), 334 (100, $[MH]^+$ (^{79}Br); *m/z* [HRMS ES+] found $[MH]^+$ 334.0446. $C_{16}H_{17}NO_2{}^{79}Br$ requires 334.0443.

Chiral HPLC was conducted on an Agilent Technologies 1260 Infinity machine with a UV detector (at 280 nm), using an Astec Chirobiotic™ T column (25 cm × 4.6 mm, 5 µm). The product (injection volume 5 µL) was eluted with MeOH (containing 0.2% AcOH and 0.1% TEA) at 1 mL/min and the (1*S*,3*S*,4*R*)-product had a retention time (r_t) of 4.3 min.

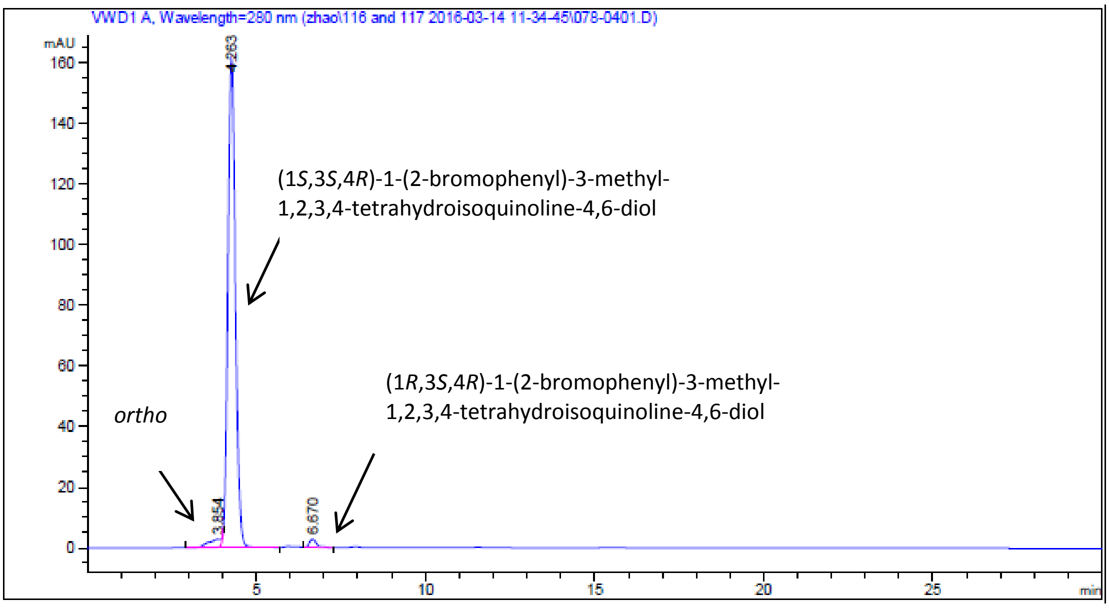

Figure 24 Product prior to recrystallisation (*de* 97%) with HPLC Astec Chirobiotic T column.

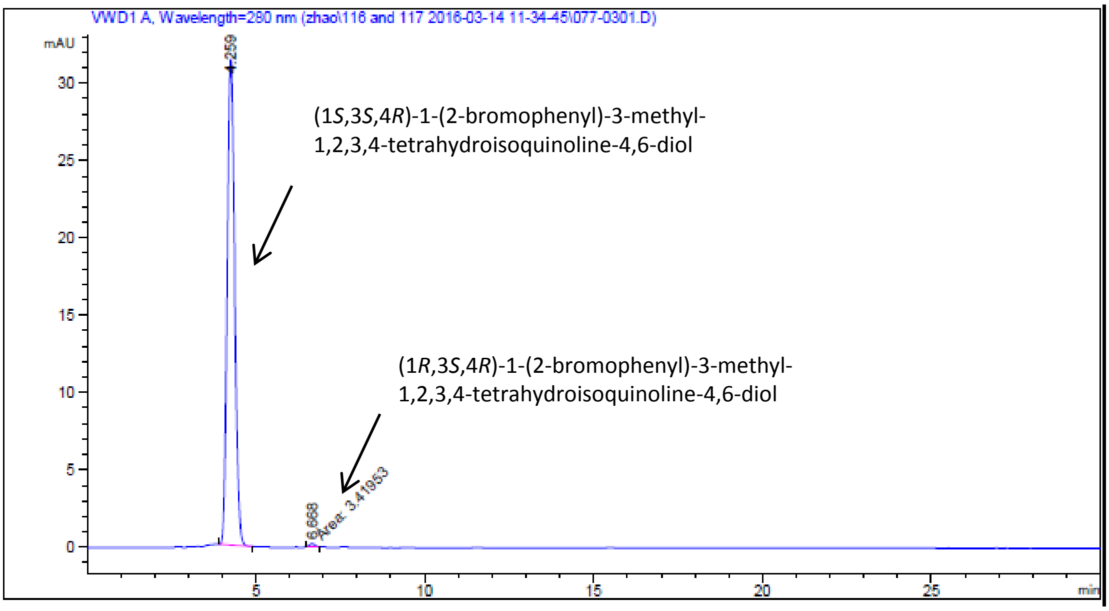

Figure 25 Product after recrystallisation (*de* 99%) with HPLC Astec Chirobiotic T column.

NMR Data

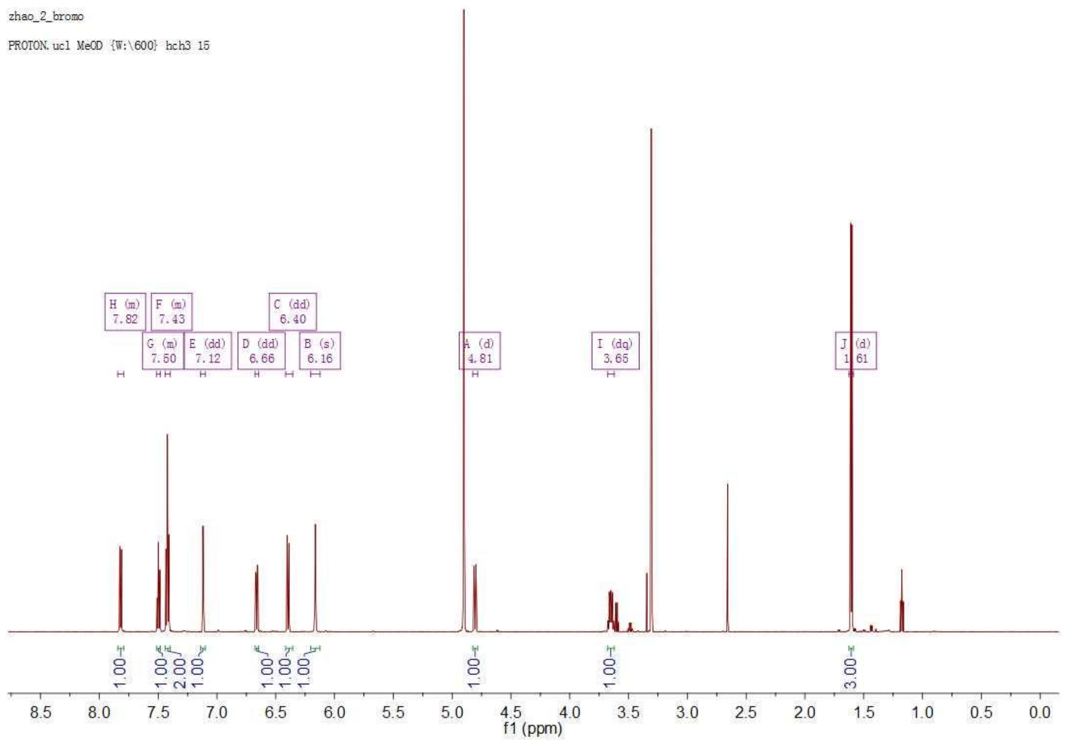

Figure 26 ^{1}H NMR.

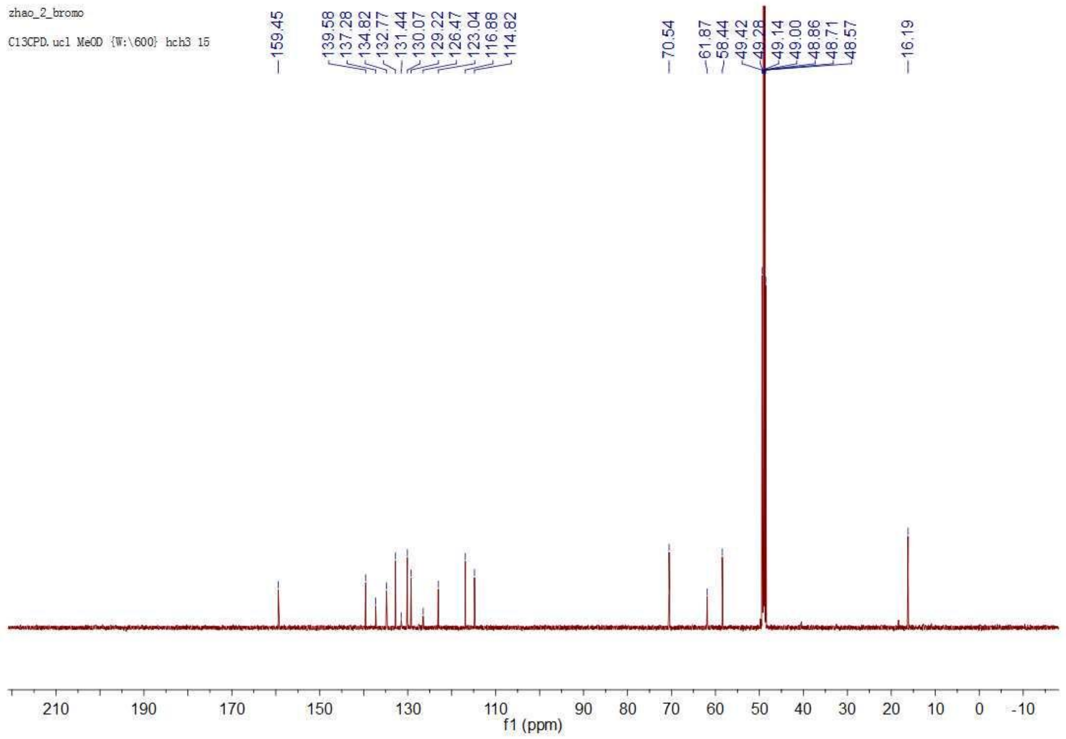

Figure 27 ^{13}C NMR.

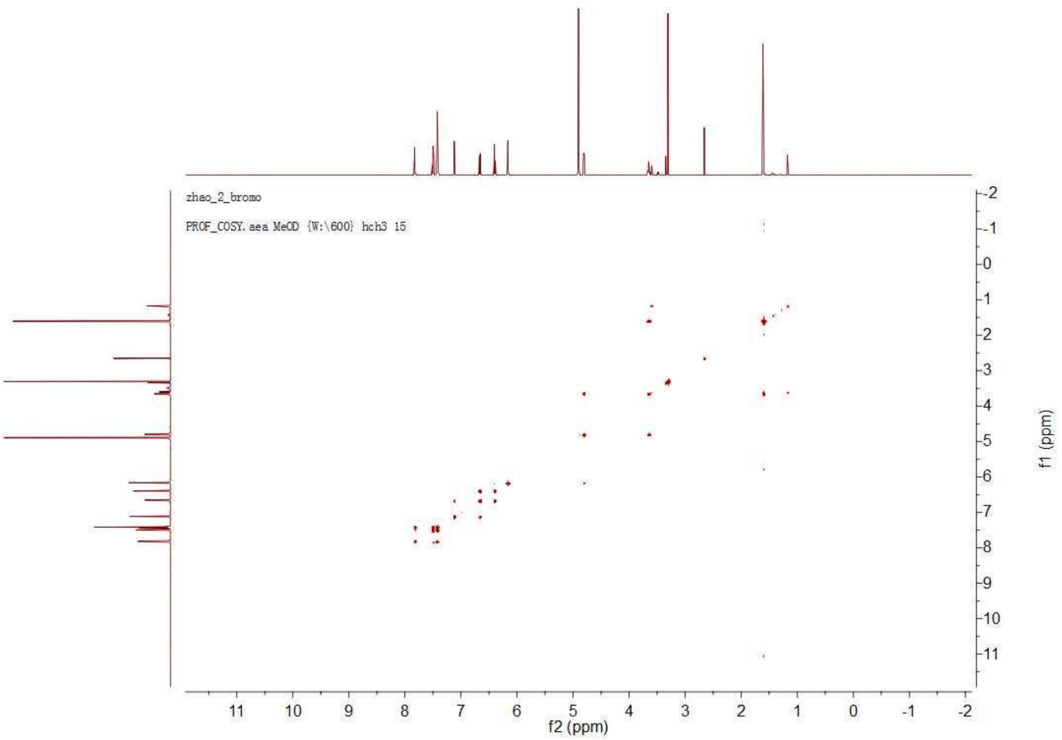

Figure 28 ^{1}H-^{1}H COSY.

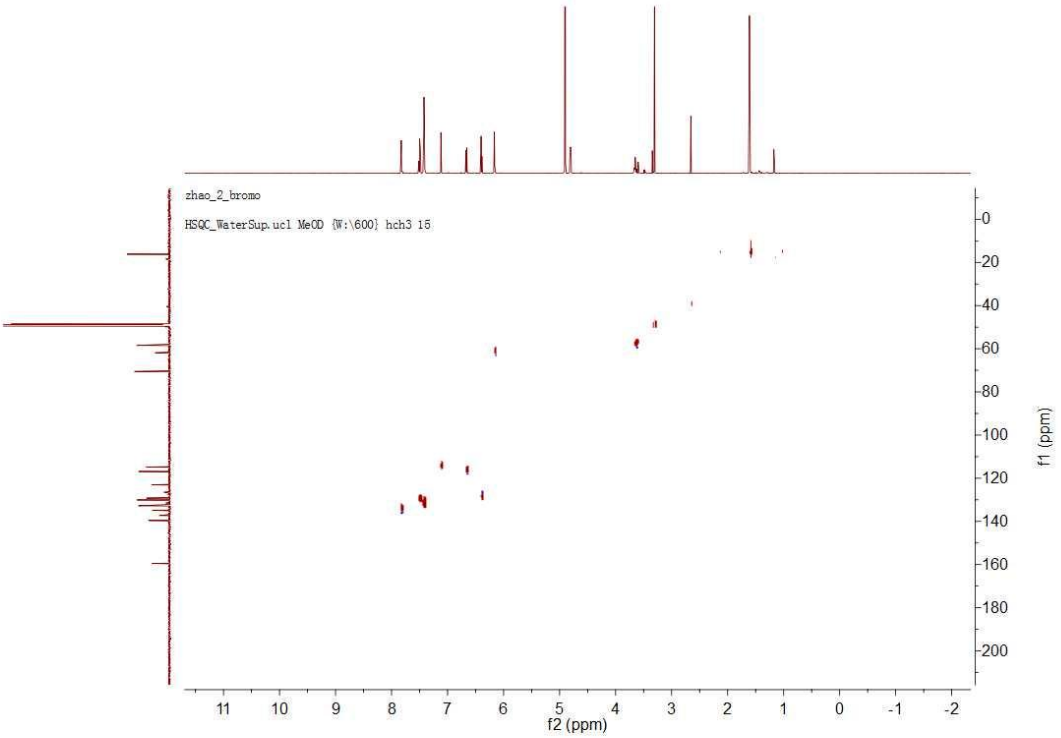

Figure 29 HMQC.

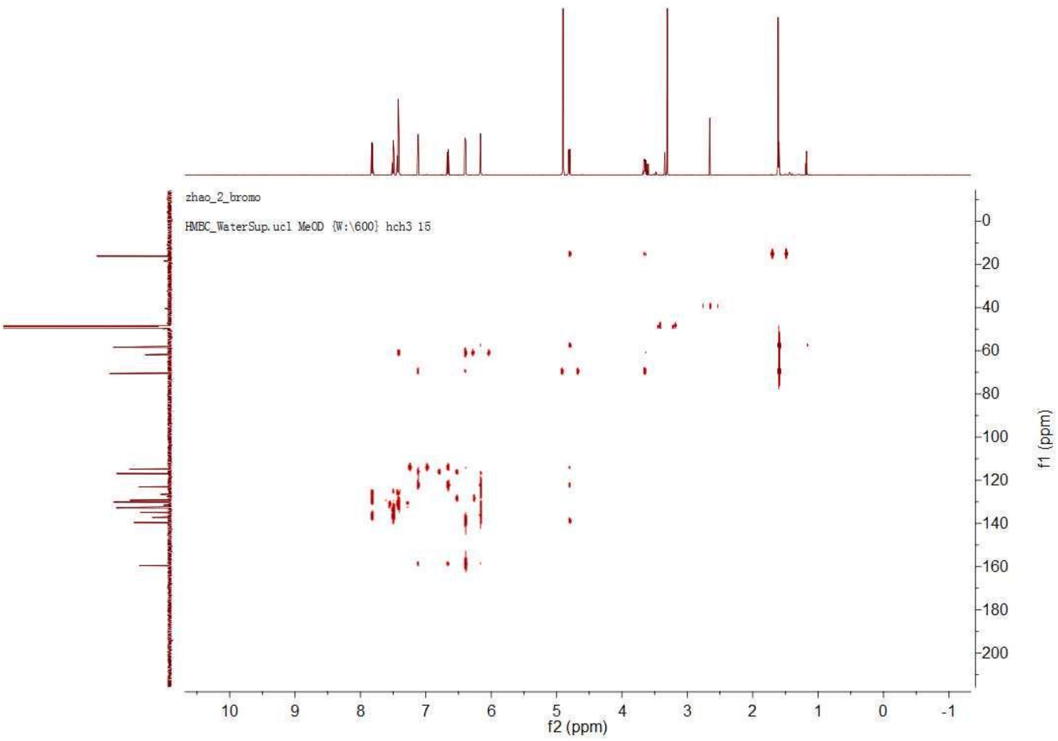

Figure 30 HMBC.

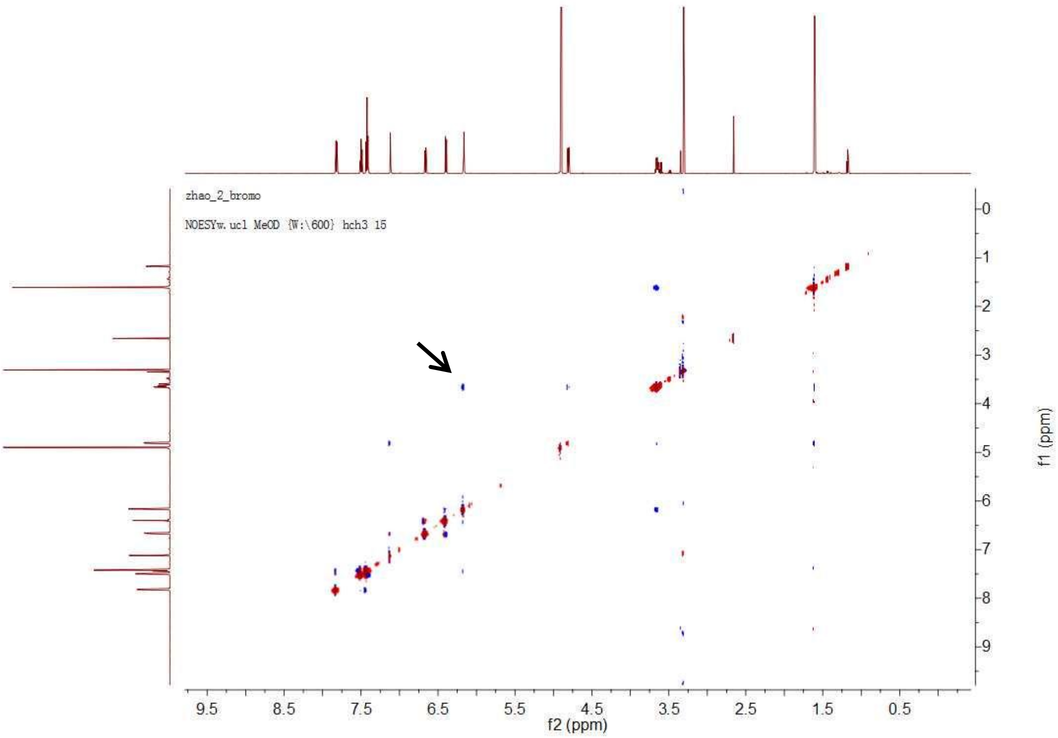

Figure 31 NOESY.

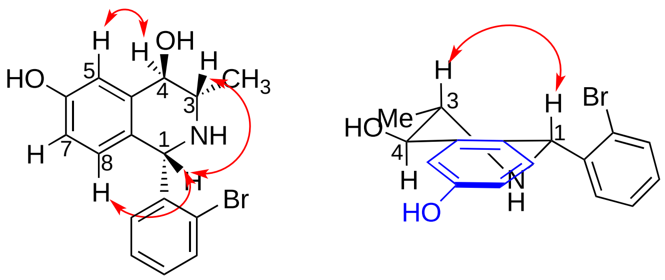

Scheme 4 An NOE was observed between 1-H and 3-H, which established that the stereochemistry at C-1 was the (*S*)-configuration (note reversal of stereocenter methyl- and hydroxy groups compared to the previous compound).

4.3 (1*S*,3*S*,4*R*)-1-(2-bromophenyl)-3-methyl-1,2,3,4-tetrahydroisoquinoline-4,6-diol synthesis under the 3-step reaction cascade conditions (3.5) to identify minor isomers formed

To (1*R*,2*S*)-metaraminol bitartrate (47.6 mg, 0.15 mmol; 15 mM) and sodium ascorbate (18.0 mg, 0.091 mmol; 10 mM), in potassium phosphate buffer (pH 7.0, 9.7 mL; 0.2 M) and DMSO (0.3 mL), 2-bromobenzaldehyde (12 μL, 0.10 mmol; 30 mM) was added. The reaction was stirred under argon at 50 °C for 24 h.

The resulting reaction mixture was directly analysed by chiral HPLC on an Agilent Technologies 1260 Infinity machine with a UV detector (at 280 nm), using an Astec Chirobiotic™ T column (25 cm × 4.6 mm, 5 μm). The product (injection volume 5 μL) was eluted with MeOH (containing 0.2% AcOH and 0.1% TEA) at 0.8 mL/min.

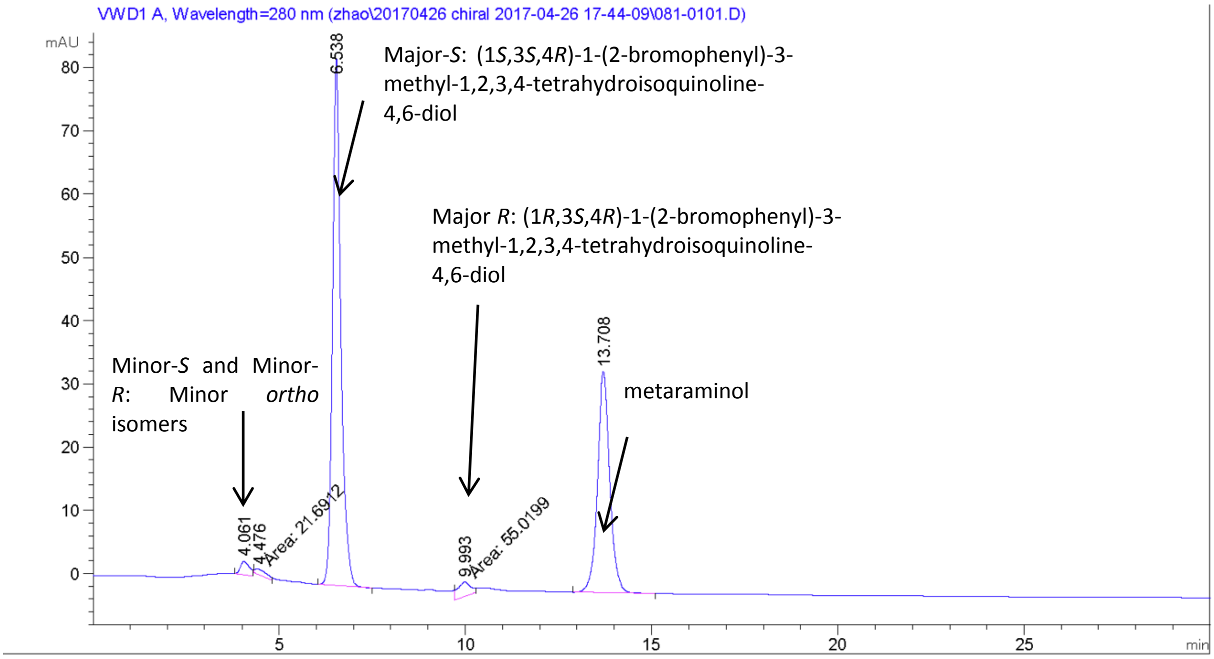

Figure 32 Analysis of the crude reaction mixture from the reaction of metaraminol with 2-bromobenzaldehyde in KPi buffer.

Table 12 HPLC data of the reaction mixture and starting materials.

Retention time [min]	Peak area	Identified compound	Ratio of percentage (calc. from HPLC)
4.1	35.6	Minor-*S* or *R*	2.5%
4.5	21.7	Minor-*S* or *R*	1.5%
6.5	1328.6	Major-*S*	92.0%
10.0	55.0	Major-*R*	4.0%
13.7	801.2	metaraminol remaining	--

Table 13 HPLC data of standard compounds.

Retention time [min]	Compound
4.2	2-bromobenzaldehyde
13.9	metaraminol

The reaction mixture was then extracted with ethyl acetate (3 × 10 mL). The organic phases were combined and washed with brine (10 mL), dried over anhydrous Na_2SO_4, filtered and concentrated under vacuum to obtain the crude product. Both the reaction mixture above and the crude product were analysed by ^{1}H NMR spectroscopy and NMR NOESY experiments.

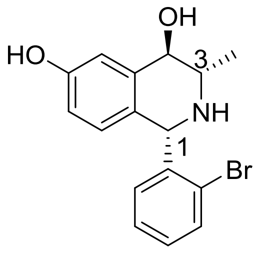

major-*S*

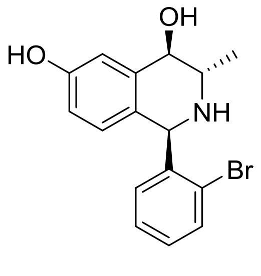

major-*R*

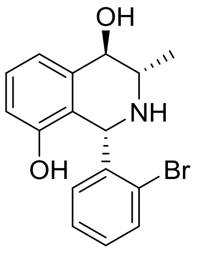

minor-*S*

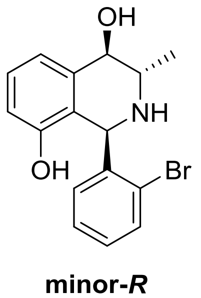

minor-*R*

Table 14 ^{1}H NMR spectroscopic data of the crude product (C-1 proton).

**Chemical shift (ppm)**	**Integration**	**Identified compound**	**Ratio of percentage (calc. from ^{1}H NMR spectra)**
5.59	13.7	Major-*S*	91%
5.54	1.0	Major-*R*	7.0%
5.48	0.13	Minor-*S* or *R*	1.0%
5.34	0.15	Minor-*S* or *R*	1.0%

This data confirms that under the KPi reaction conditions for the final step, the major-(1*S*,3*S*,4*R*)-isomer is formed with some (1*R*,3*S*,4*R*)-isomer (termed here major-*R*) in a ratio of 96:4 by chiral HPLC. In addition a small amount of the *ortho*-product (termed here minor-*S* or *R*) was produced <4%.

NMR data

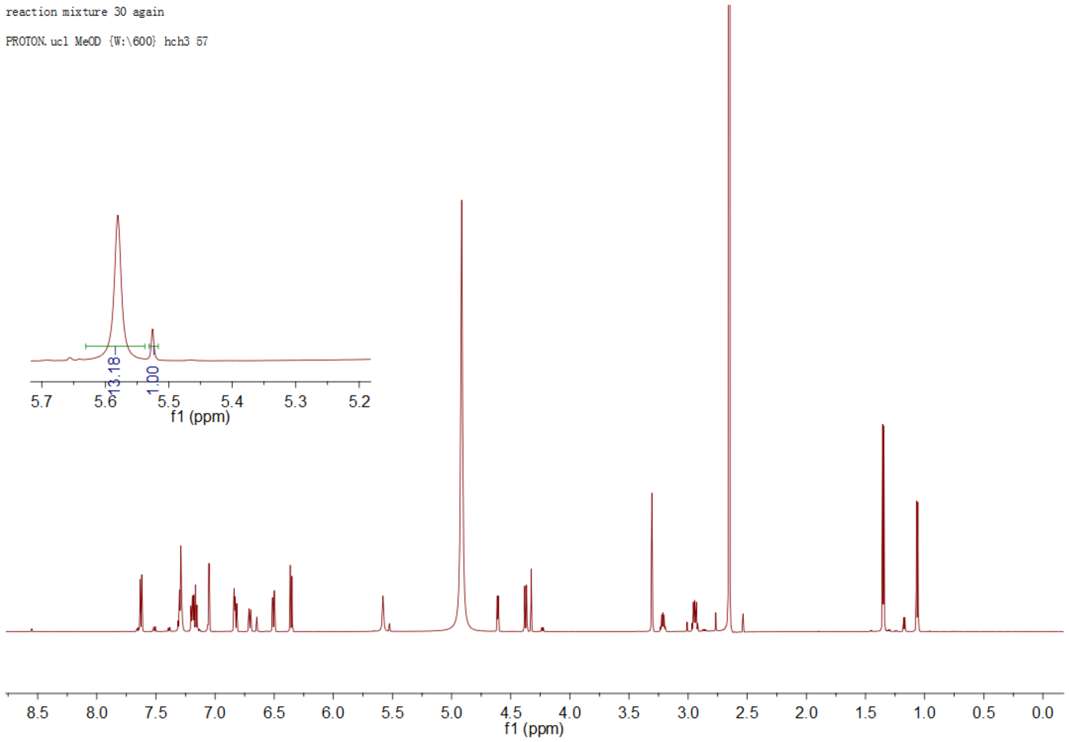

Figure 33 ^{1}H NMR of reaction mixture.

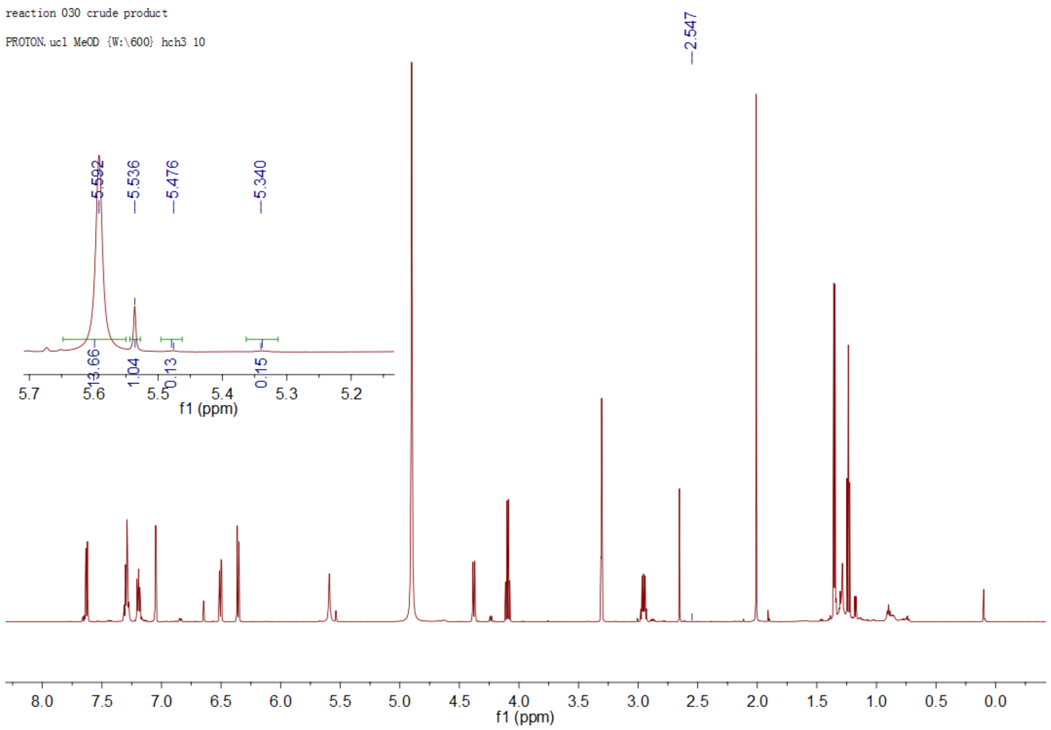

Figure 34 ^{1}H NMR spectrum of the crude product: the NMR of the crude product has slightly different shifts to the purified product as the latter was an HCl salt.

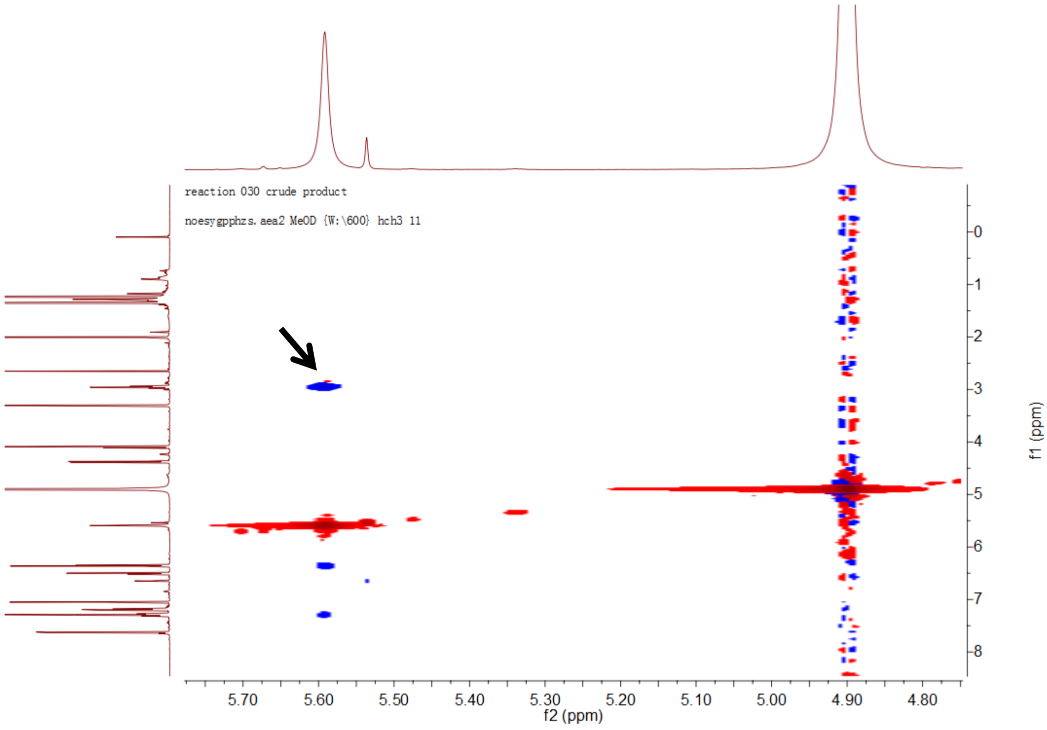

Figure 35 NOESY: NOE observed between C-1 proton and C-3 proton (compound major-*S*).

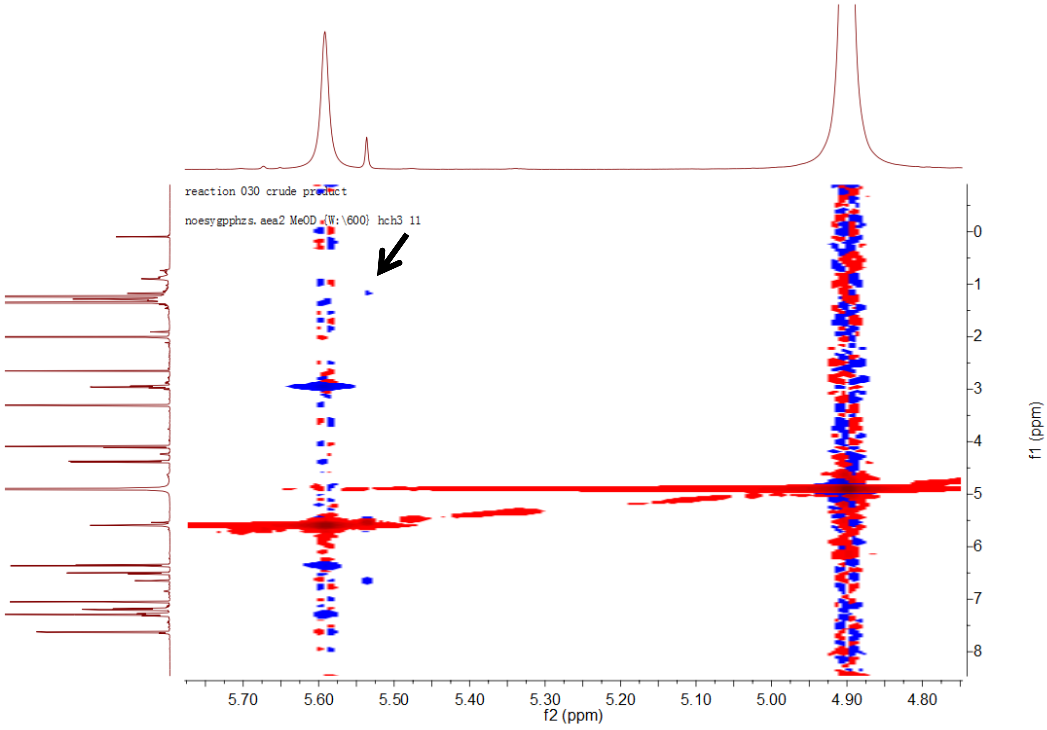

Figure 36 NOESY: NOE observed between C-1 proton and C-3 methyl group (compound major-*R*).

5. Calculation of conversion, yield, *ee*, *de*, *dc* and *ic* values

Total conversion [%] = [substrate$_{t=0}$]*relative conversion (S1)*relative conversion (S2)*relative conversion (S3)

Total *ic* [%] = 100*relative *ic* (S1)*relative *ic* (S2)*relative *ic* (S3)

Conversion [%] = ([substrate$_t$] / ([product$_{t=0}$] + [substrate$_{t=0}$])) * 100

Yield* [%] = ([product]$_t$ / ([product]$_{t=0}$ + [substrate]$_{t=0}$) * 100

enantiomeric excess ee [%] = ((E1 - E2) / (E1 + E2)) * 100

diastereomeric excess de [%] = ((D1 – (D2 + D3)) / (D1 + D2 + D3)) * 100

diastereomeric content dc [%] = (D1 / ($\sum D_i$)) * 100

isomeric content[13] *ic* [%] = (I1 / ($\sum I_i$)) * 100

E: enantiomer, D: diastereomer, I: isomer; $\sum I_i$: sum of all isomers in the mixture, S: cascade step

*non-isolated (determined *via* instrumental product analysis)

6. Conversion of 2-amino-1-(3-hydroxyphenyl)propan-1-ol isomers by norcoclaurine synthase variants and phosphate (step 3)

The following NCS were screened against all four isomers of 2-amino-1-(3-hydroxyphenyl)propan-1-ol and phenylacetaldehyde for the enzymatic (NCS) and chemically (phosphate) catalysed Pictet-Spengler reaction. Three of the four 2-amino-1-(3-hydroxyphenyl)propan-1-ol isomers are possible candidates for Pictet-Spengler reaction towards 1-benzyl-3-methyl-1,2,3,4-tetrahydroisoquinoline-4,6-diol catalysed by NCS ((1*R*,2*S*)-, (1*R*,2*R*)- and (1*S*,2*R*)-2-amino-1-(3-hydroxyphenyl)propan-1-ol). *Tf*NCS-A79I was identified as best catalyst for the enzyme catalysed conversion of (1*R*,2*S*)- and (1*S*,2*S*)-2-amino-1-(3-hydroxyphenyl)propan-1-ol. Only the (1*S*,2*R*)-isomer was not accepted by any of the screened NCS. As background reaction, potassium phosphate catalyses the cyclisation of three of the four 3-hydroxyl amino alcohols. The (1*S*,2*S*)-stereoisomer is not converted by potassium phosphate, but yielding in the presence of phenylacetaldehyde and NCS the enzymatic cyclisation product. These results underline that further THIQ isomers are accessible by the described method as well.

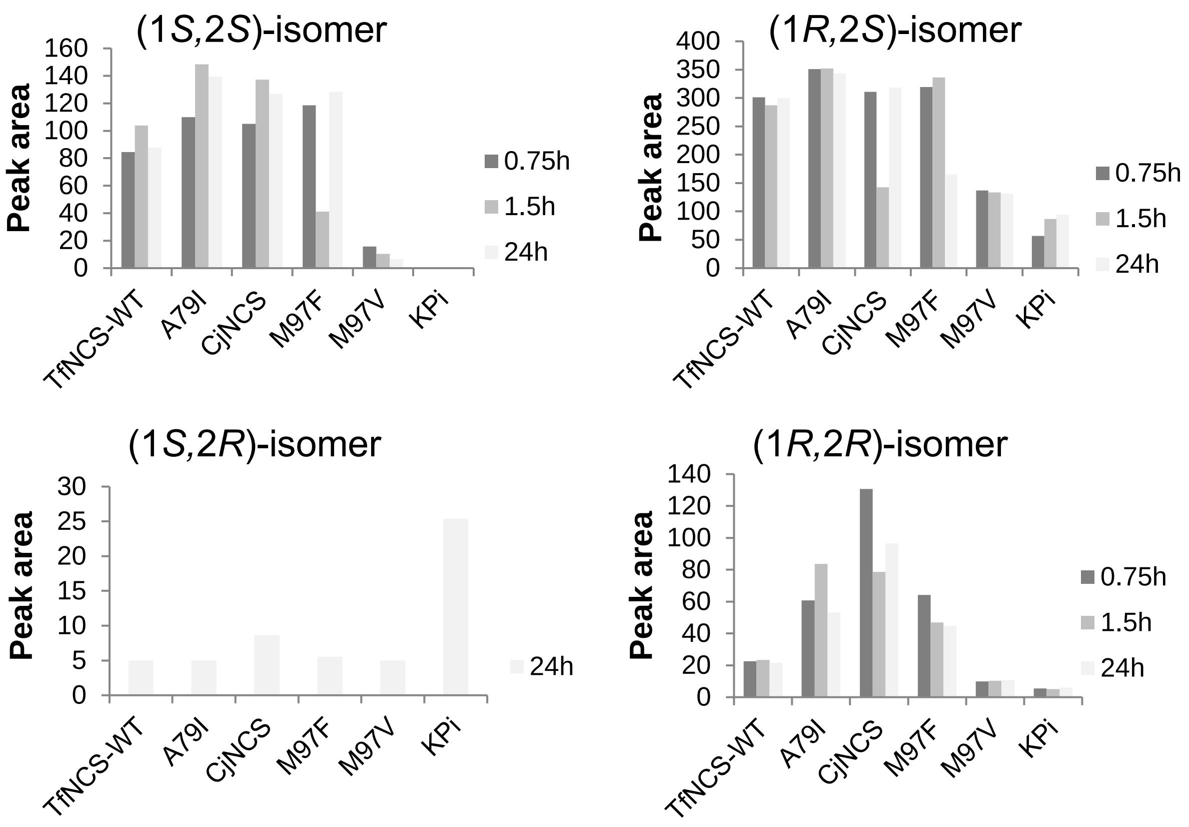

Figure 37 Syntheses of different 1-benzyl-3-methyl-1,2,3,4-tetrahydroisoquinoline-4,6-diol isomers starting from the four 2-amino-1-(3-hydroxyphenyl)-propane-1-ol isomers. Reaction conditions: 8.5 mM metaraminol, 9.25 % DMSO, 15 mM phenylacetaldehyde, 0.5 mg/mL purified and lyophilised enzyme, for KPi control: 100 mM pH 7, *Tf*NCS-wt: wildtype norcoclaurine synthase of *Thalictrum flavum*, and three variants thereof, *Cj*NCS: *Coptis japonica* norcoclaurine synthase.

7. Substrate screening for phosphate catalysed Pictet-Spengler reaction (step 3)

A substrate screen for most suitable substrates in terms of good conversion and highest stereoselectivity for the phosphate catalysed Pictet-Spengler reaction was performed. Product peaks were tentatively assigned, as chemical standards were not available at the time.

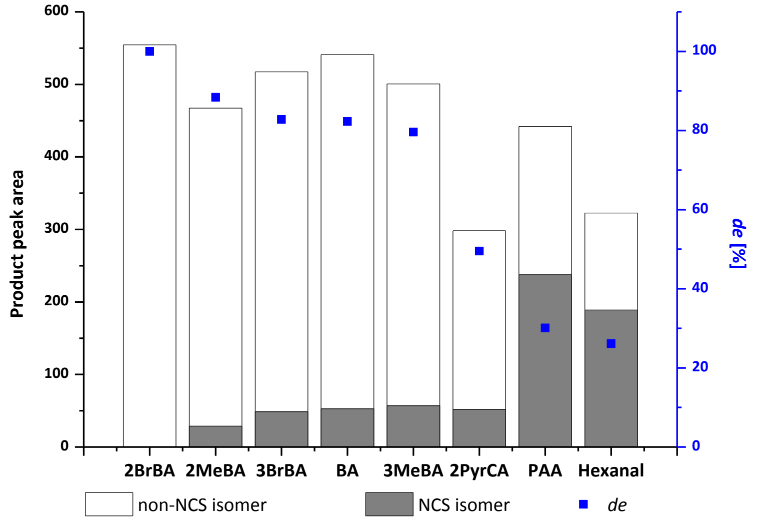

Figure 38 Substrate screening of the phosphate catalysed Pictet-Spengler reaction of commercially available (1*R*,2*S*)-2-amino-1-(3-hydroxyphenyl)propan-1-ol towards THIQs. Product peak area determined according to HPLC-analytics. BA: benzaldehyde, 2BrBA: 2-bromobenzaldehyde, 3BrBA: 3-bromobenzaldehyde, 2MeBA: 2-methylbenzaldehyde, 3MeBA: 3-methylbenzaldehyde, 2PyrCA: 2-pyridinecarboxaldehyde, PAA: phenylacetaldehyde, *de*: diastereomeric excess (single measurements); reaction conditions: total volume of 1 mL, 800 mM KPi buffer, pH 7.0, 10 mM (1*R*,2*S*)-metaraminol (commercially available), 10 mM aldehyde, 10% DMSO, 50 °C, 500 rpm, 1 h, PAA: changed reaction conditions: 8.5 mM (1*R*,2*S*)-metaraminol, 15 mM PAA, 100 mM, pH 7.0 KPi, 5% DMSO).

8. Time curve of the enzymatic 3-step cascade reaction to (1*S*,3*S*,4*R*)-1-benzyl-3-methyl-1,2,3,4-tetrahydroisoquinoline-4,6-diol (step 1-3)

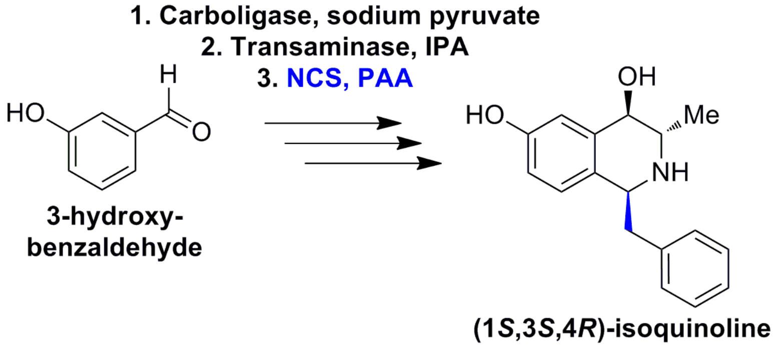

Scheme 5 Overview of enzymatic 3-step cascade to (1*S*,3*S*,4*R*)-1-benzyl-3-methyl-1,2,3,4-tetrahydroisoquinoline-4,6-diol (IPA: isopropylamine, NCS: norcoclaurine synthase, PAA: phenylacetaldehyde).

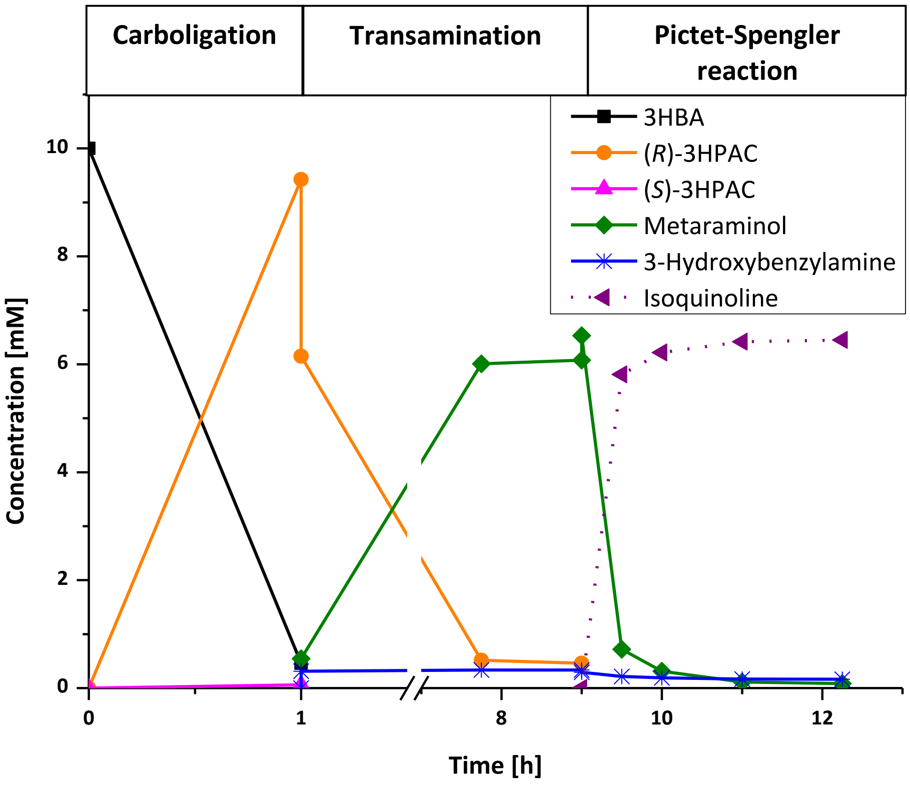

Figure 39 Reaction process of a typical 3 step cascade reaction.

A typical reaction process curve of the enzymatic 3-step cascade from 3-hydroxybenzaldehyde and sodium pyruvate *via* the intermediates (*R*)-3HPAC and (1*R*,2*S*)-metaraminol to the end product (1*S*,3*S*,4*R*)-1-benzyl-3-methyl-1,2,3,4-tetrahydroisoquinoline-4,6-diol is shown.

Reaction conditions are described in SI-2. All substrate and product concentrations according to instrumental analytics are shown, except for the isoquinoline product. As there was no calibration of the isoquinoline end product feasible, the conversion was calculated from 2-amino-1-(3-hydroxyphenyl)propan-1-ol (metaraminol) depletion over time. As the (1*S*,3*S*,4*R*)-isoquinoline was the only product of the Pictet-Spengler reaction, measured by HPLC (Fig. 8d), we transferred the substrate decrease of metaraminol directly to the assumed product increase of (1*S*,3*S*,4*R*)-isoquinoline ( ) in this figure.

9. Literature

[1] B. R. Lichman, J. Zhao, H. C. Hailes, J. M. Ward, *Nat. Commun.* **2017**, *8*, 14883.

[2] B. R. Lichman, E. D. Lamming, T. Pesnot, J. M. Smith, H. C. Hailes, J. M. Ward, *Green Chem.* **2015**, *17*, 852–855.

[3] O. Weinstock, C. Sella, D. M. Chipman, Z. Barak, *J. Bacteriol.* **1992**, *174*, 5560–5566.

[4] M. Vyazmensky, C. Sella, Z. Barak, D. M. Chipman, *Biochemistry* **1996**, *35*, 10339–10346.

[5] V. Vinogradov, M. Vyazmensky, S. Engel, I. Belenky, A. Kaplun, O. Kryukov, Z. Barak, D. M. Chipman, *Biochim. Biophys. Acta* **2006**, *1760*, 356–363.

[6] U. Schell, R. Wohlgemuth, J. M. Ward, *J. Mol. Catal. B Enzym.* **2009**, *59*, 279–285.

[7] U. Kaulmann, K. Smithies, M. E. B. Smith, H. C. Hailes, J. M. Ward, *Enzyme Microb. Technol.* **2007**, *41*, 628–637.

[8] T. Sehl, R. C. Simon, H. C. Hailes, J. M. Ward, U. Schell, M. Pohl, D. Rother, *J. Biotechnol.* **2012**, *159*, 188–194.

[9] T. Sehl, H. C. Hailes, J. M. Ward, R. Wardenga, E. von Lieres, H. Offermann, R. Westphal, M. Pohl, D. Rother, *Angew. Chem. Int. Ed.* **2013**, *52*, 6772–6775.

[10] J. J. Maresh, S. O. Crowe, A. A. Ralko, M. D. Aparece, C. M. Murphy, M. Krzeszowiec, M. W. Mullowney, *Tetrahedron Lett.* **2014**, *55*, 5047–5051.

[11] N. Paczia, A. Nilgen, T. Lehmann, J. Gätgens, W. Wiechert, S. Noack, *Microb. Cell Fact.* **2012**, *11*, 122.

[12] J. Hummel, J., Strehmel, N., Selbig, J., Walther, D. and Kopka, *Metabolomics* **2010**, *6*, 322–333.

[13] J. Wachtmeister, A. Jakoblinnert, D. Rother, *Org. Process Res. Dev.* **2016**, *20*, 1744–1753.
